# Intergenic *Alu* exonisation facilitates the evolution of tissue-specific transcript ends

**DOI:** 10.1093/nar/gkv956

**Published:** 2015-09-22

**Authors:** Mojca Tajnik, Alessandra Vigilante, Simon Braun, Heike Hänel, Nicholas M. Luscombe, Jernej Ule, Kathi Zarnack, Julian König

**Affiliations:** 1 MRC Laboratory of Molecular Biology, Francis Crick Avenue, Cambridge Biomedical Campus, Cambridge CB2 0QH, UK; 2 International Centre for Genetic Engineering and Biotechnology, Padriciano 99, 34149 Trieste, Italy; 3 UCL Genetics Institute, Department of Genetics, Evolution & Environment, University College London, Gower Street, London WC1E 6BT, UK; 4 Cancer Research UK London Research Institute, 44 Lincoln's Inn Fields, London WC2A 3LY, UK; 5 Institute of Molecular Biology (IMB) gGmbH, Ackermannweg 4, 55128 Mainz, Germany; 6 Okinawa Institute of Science & Technology, 1919-1 Tancha, Onna-son, Kunigami-gun, Okinawa 904-0495, Japan; 7 Department of Molecular Neuroscience, UCL Institute of Neurology, Queen Square, London WC1N 3BG, UK; 8 Buchmann Institute for Molecular Life Sciences (BMLS), Max-von-Laue-Str. 15, 60438 Frankfurt, Germany

## Abstract

The 3′ untranslated regions (3′ UTRs) of transcripts serve as important hubs for posttranscriptional gene expression regulation. Here, we find that the exonisation of intergenic *Alu* elements introduced new terminal exons and polyadenylation sites during human genome evolution. While *Alu* exonisation from introns has been described previously, we shed light on a novel mechanism to create alternative 3′ UTRs, thereby opening opportunities for differential posttranscriptional regulation. On the mechanistic level, we show that intergenic *Alu* exonisation can compete both with alternative splicing and polyadenylation in the upstream gene. Notably, the *Alu*-derived isoforms are often expressed in a tissue-specific manner, and the *Alu*-derived 3′ UTRs can alter mRNA stability. In summary, we demonstrate that intergenic elements can affect processing of preceding genes, and elucidate how intergenic *Alu* exonisation can contribute to tissue-specific posttranscriptional regulation by expanding the repertoire of 3′ UTRs.

## INTRODUCTION

The 3′ untranslated regions (3′ UTRs) of transcripts are important for gene expression regulation. More than 50% of human genes give rise to multiple isoforms with alternative 3′ UTRs, thereby enabling tissue-specific or developmental regulation of transcripts that otherwise encode for the same protein function (1). A prevalent mechanism to generate alternative 3′ UTRs is the usage of alternative terminal exons and polyadenylation signals (PASs) that trigger endonucleolytic cleavage and addition of the polyadenosine (polyA) tail. Despite their importance however, the evolution of alternative 3′ UTRs has received little attention to date.

Transposable elements (TEs) are major driving forces for human genome evolution. The most abundant class of TEs are the primate-specific *Alu* elements that comprise more than 10% of the human genome (2). When inserted in antisense orientation into a transcribed region, *Alu* elements harbour multiple cryptic splicing signals. Previous studies suggest that 5% of all internal alternative exons in the human genome originated from activation of these cryptic signals in a process called *Alu* exonisation (3,4). Moreover, the uncontrolled inclusion of intronic *Alu* elements as cryptic exons has been associated with several human diseases (5,6). However, whereas considerable attention has been paid to the interference of intronic *Alu* elements with splicing, the effect of *Alu* elements in the intergenic regions in the neighbourhood of genes remained largely unexplored.

Here, we show how the exonisation of intergenic *Alu* elements located downstream of genuine polyadenylation sites can affect the 3′ end processing of transcripts. Using genome-wide assays, comparative genomics and minigene experiments, we provide evidence for a kinetic competition between intergenic *Alu* exonisation and polyadenylation or splicing in the preceding gene. We also show tissue-specific regulation of the newly formed 3′ ends. Our study presents a previously undescribed mechanism to generate new alternative 3′ ends during human genome evolution.

## MATERIALS AND METHODS

### Cell culture, silencing of *HNRNPC* and reporter minigene transfections

HeLa, CAL-51, Hep G2 and HEK293T cells were grown in Dulbecco's modified Eagle medium (DMEM) supplemented with 10% fetal bovine serum and 1% penicillin–streptomycin and cultured at 37°C with 5% CO_2_. Depletion of heterogeneous nuclear ribonucleoproteins C1/C2 (hnRNP C) and reporter minigene transfections were performed as previously described (7). Briefly, HeLa cultures were independently transfected using two different hnRNP C Stealth Select RNAi siRNAs (KD1 and KD2 refer to siRNAs HSS179304 and HSS179305 from Invitrogen, respectively) at a final concentration of 5 nM. Western blot analyses confirmed efficient *HNRNPC* knockdown with both siRNAs without affecting U2AF65 protein levels. Following *HNRNPC* knockdown transfections, cells were cotransfected with 400 ng of minigene plasmids. Cultures were harvested 48 h later and RNA was extracted for downstream experiments. All transfection experiments were performed in triplicates.

### Data analysis

Data analysis was performed using R-3.0.2 (R Core Team). The R packages ggplot2 (0.9.3), plyr (1.8), reshape2 (1.2.2) and the Bioconductor package GenomicRanges (1.14.3) were used throughout the analysis.

### iCLIP and RNA-seq

We used high-throughput sequencing data obtained from our previous experiments (7,8). In particuar, RNA-seq experiments had been performed on two replicate samples from two *HNRNPC* knockdowns (KD1 and KD2) as well as from control HeLa cells. ArrayExpress accession numbers for the previously published RNA-seq and iCLIP data are E-MTAB-1147 and E-MTAB-1371, respectively.

We used the list of 1875 *Alu* exons obtained in (7). Briefly, we had used the splice-aware algorithm TopHat (version 1.1.4; relevant parameters: -min-isoform-fraction 0 -coverage-search) (9) to align the RNA-seq reads to the human genome (hg19) and Cufflinks (version 0.9.3, -min-isoform-fraction 0 to detect weakly included exons) (10) to predict exons from the collapsed RNA-seq data. The *Alu* exons had then been identified by postprocessing Cufflinks exon predictions according to the following criteria: (i) all *Alu* exons had to show at least one splice site within an *Alu* element that was supported by junction-spanning reads, and (ii) the exon predictions had to be unambiguous, i.e. no other predicted exons should overlap with the *Alu* exons. In order to identify *Alu* exons in intergenic regions, we considered the set of polyA sites from Ensembl database (version 67) and classified all *Alu* exons downstream of the last genuine polyadenylation site of the respective genes as intergenic *Alu* exons. We further calculated the distance between the last polyA site and the associated *Alu* exon.

**Figure 1.**
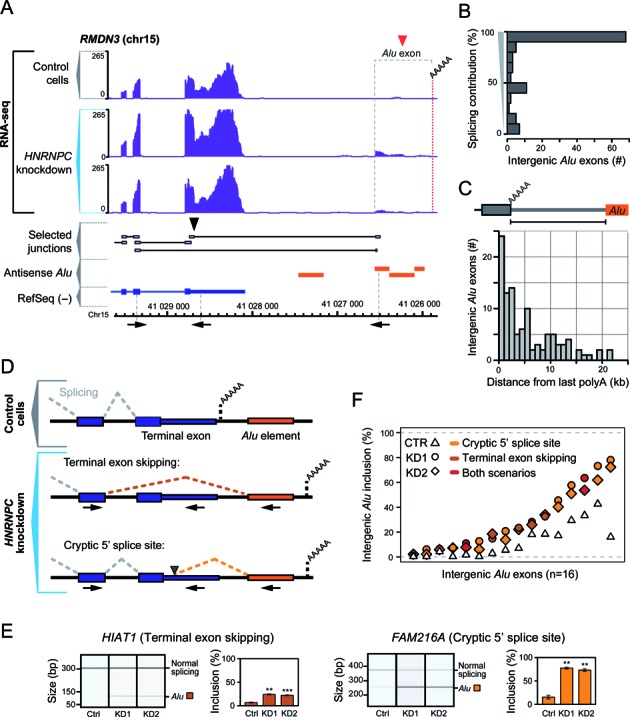
Intergenic *Alu* elements downstream of genuine polyadenylation sites can exonise in the absence of hnRNP C. (**A**) Genome browser view of the *RMDN3* gene and surrounding intergenic regions (chr15, nt 41 025 807–41 029 604, minus strand), displaying the RNA-seq data (reads per nucleotide; purple) from control and *HNRNPC* knockdown HeLa cells. The red and black arrowheads mark the hnRNP-C-repressed intergenic *Alu* exon and the usage of a cryptic 5′ splice site within the terminal exon, respectively. RefSeq transcript annotations (blue), selected RNA-seq junction-spanning reads (grey) and *Alu* elements in antisense orientation to the shown strand (orange) are depicted below. The dashed red line indicates the new polyadenylation site that is used upon intergenic *Alu* exonisation, as determined by 3′ RACE. The positions of the primers used for RT-PCR validations are depicted below (not drawn to scale). (**B**) Bar diagram depicting the number of intergenic *Alu* exons which show a given level of splicing contribution. Displayed is the fraction of junction-spanning reads versus total reads overlapping the 3′ splice sites of the *Alu* exons. (**C**) Bar diagram depicting the distances (in kb) between the intergenic *Alu* elements and the last polyadenylation sites of the 107 corresponding genes, for which we observe intergenic *Alu* exonisation in the absence of hnRNP C. (**D**) Schematic representation of the two scenarios of intergenic *Alu* exonisation. Under control conditions, there is splicing of the terminal exon and polyadenylation occurring at the genuine polyadenylation site of the gene (top). In absence of hnRNP C, intergenic *Alu* exonisation causes skipping of the complete terminal exon (middle) and/or splicing at a cryptic 5′ splice site within the terminal exon (bottom). In both cases, polyadenylation occurs at a new site within or downstream of the intergenic *Alu* element (orange). The positions of the primers used for RT-PCR validations (Figure 1E, F, Supplementary Figures S2 and S3) are depicted below the two *Alu* exonisation scenarios. (**E**) Semiquantitative RT-PCR monitoring intergenic *Alu* exonisation in two example genes employing terminal exon skipping (left) or activation of a cryptic 5′ splice site within the terminal exon (right). *Alu* exon inclusion was measured in control (Ctrl) and *HNRNPC* knockdown (KD1 and KD2) HeLa cells. Shown are gel-like representations of capillary electrophoresis and quantification of average *Alu* exon inclusion levels. Asterisks represent different significance levels when compared to control conditions (**P value < 10^−3^; ***P < 10^−4^; Student's *t*-test). Error bars represent standard deviation of the mean, n = 3. (**F**) Plot depicting the mean inclusion levels (in %) in control HeLa cells (CTR, white triangles) and both *HNRNPC* knockdowns (KD1, filled circles; KD2, filled diamonds) of 16 intergenic *Alu* exons that were validated by semiquantitative RT-PCR (Supplementary Figures S2 and S3). The light orange, dark orange and red colours represent cryptic 5′ splice site activation, terminal exon skipping and co-occurrence of both scenarios, respectively.

To assess the relative contribution of splicing-mediated *Alu* exon inclusion, we extracted all reads overlapping the 3′ splice site of the *Alu* exon (i) by at least 10 nt on either side of the 3′ splice site in the case of continously aligning reads, or (ii) continuing across the predicted exon-exon junction in the case of junction-spanning reads (defined by TopHat) (9). We then calculated the fraction of junction-spanning versus total overlapping reads as a proxy of the relative splicing contribution (Figure 1B).

Gene Ontology (GO) analysis was performed using the DAVID tool (11) using the genes containing intergenic *Alu* exons as input list and all other genes in the genome as background. Results from functional annotation analysis were considered applying a *P* value cut-off at 0.05, and used as input for the ReviGO visualization tool (12). We used a graph-based visualization obtaining the XGMML file hence imported and modified in Cytoscape (13).

### RT-PCR quantification of intergenic *Alu* exons

In order to validate the splicing changes of identified intergenic *Alu* exons from our RNA-seq data in control and *HNRNPC* knockdown conditions, we used semiquantitative RT-PCR (Figure 1E, F, Supplementary Figures S2 and S3). For this, we extracted total RNA from transfected HeLa cells. Total RNA was reverse transcribed using the RevertAid™ Premium First Strand cDNA Synthesis Kit (Fermentas), to obtain a template for semiquantitative RT-PCR reactions using IMMOLASE™ DNA Polymerase (Bioline). The conditions were as follows: 95°C for 10 min, 35 cycles of [95°C for 10 s, 55°C for 10 s, 72°C for 30 s] and then final extension at 72°C for 2 min. For each intergenic *Alu* exon, we used a forward oligonucleotide annealing to the upstream constitutive exon and reverse oligonucleotides annealing to both the intergenic *Alu* exon and the final region of the terminal exon (Supplementary Table S2). Using this approach we could differentiate whether the exonised intergenic *Alu* element was included after terminal exon skipping and/or there were cryptic 5′ splice sites used within the terminal exon. A QIAxcel capillary gel electrophoresis system (Qiagen) was used to visualize the PCR products and quantify each isoform. All measurements were performed in triplicates.

### Reporter minigene assays

Selected genomic loci were PCR-amplified from genomic DNA using Phusion High-Fidelity DNA Polymerase (NEB). All minigene constructs are designed with cloning the HindIII and XhoI-digested PCR products into expression vector pcDNA3 (Invitrogen), opened with the same enzymes. The obtained constructs were sequenced in order to verify that the respective sequence is identical to the expected, either in wild-type and mutated contructs.

For the *SAFB* wild-type minigene (SAFB WT), the respective genomic region was PCR-amplified from genomic DNA using SAFB_WT_F and SAFB_WT_R oligonucleotides (Supplementary Table S4). The PCR product was digested and ligated into pcDNA3. Mutations in the upstream polypyrimidine tract of the *Alu* element were introduced through PCR with the oligonucleotides SAFB_WT_F with SAFB_PPT1_R and SAFB_WT_R with SAFB_PPT1_F on linearized SAFB WT plasmid DNA. After the third PCR with the outer oligonucleotides, the product was cut and inserted into pcDNA3 to obtain the SAFB PPT1 minigene. Using the same approach, the plasmid SAFB PPT2 with mutations in the linker uridine tract was created using the oligonucleotides SAFB_PPT2_F and SAFB_PPT2_R. In order to mutate the polyadenylation signal (PAS), we introduced a single nucleotide mutation into the PAS using SAFB_PAS_F and SAFB_PAS_R on linearized SAFB WT minigene. After the third PCR with the outer oligonucleotides, the product was digested and ligated into pcDNA3 to obtain the SAFB PAS minigene.

For the *RMDN3* wild-type reporter minigene (RMDN3 WT), the respective genomic region was amplified from human genomic DNA using oligonucleotides RMDN3_WT_F and RMDN3_WT_R. The product was cut and ligated into pcDNA3. For the upstream polypyrimidine tract-mutated minigene (RMDN3 PPT1), the single nucleotide substitutions were introduced by PCR on linearized RMDN3 WT using RMDN3_PPT1_F and RMDN3_PPT1_R. After the final PCR using outer oligonucleotides, the product was cut and ligated into the vector. Using the same approach, we obtained minigenes with the mutated 5′ splice site of the *Alu* element (RMDN3 5′SS) using RMDN3_5′SS_F and RMDN3_5′SS_R and the minigene with the mutated PAS (RMDN3 PAS) using RMDN3_PAS_F and RMDN3_PAS_R.

The *C19ORF60* wild-type minigene (C19ORF60 WT) was designed with cloning the PCR-amplified genomic locus using C19orf60_WT_F and C19orf60_WT_R, digestion of the product and ligation into pcDNA3. Mutations in the upstream polypyrimidine tract of the *Alu* element (C19ORF60 PPT1) were done with a 3-step PCR approach using C19orf60_PPT1_F, C19orf60_PPT1_R and outer oligonucleotides and similar for the linker uridine tract (C19ORF60 PPT2) using C19orf60_PPT2_F, C19orf60_PPT2_R together with the outer oligonucleotides for the final PCR. We linearized C19ORF60 PPT1 minigene with BglII and used the same oligonucleotides as for C19ORF60 PPT2 to obtain the minigene with the mutations in both polypyrimidine tracts (C19ORF60 PPT1+PPT2). Moreover, the constructed minigene with depleted the whole *Alu* element sequence (C19ORF60 noAlu) was created on linearized C19ORF60 WT using C1960_noAlu_F, C1960_noAlu_R and outer oligonucleotides.

**Figure 2.**
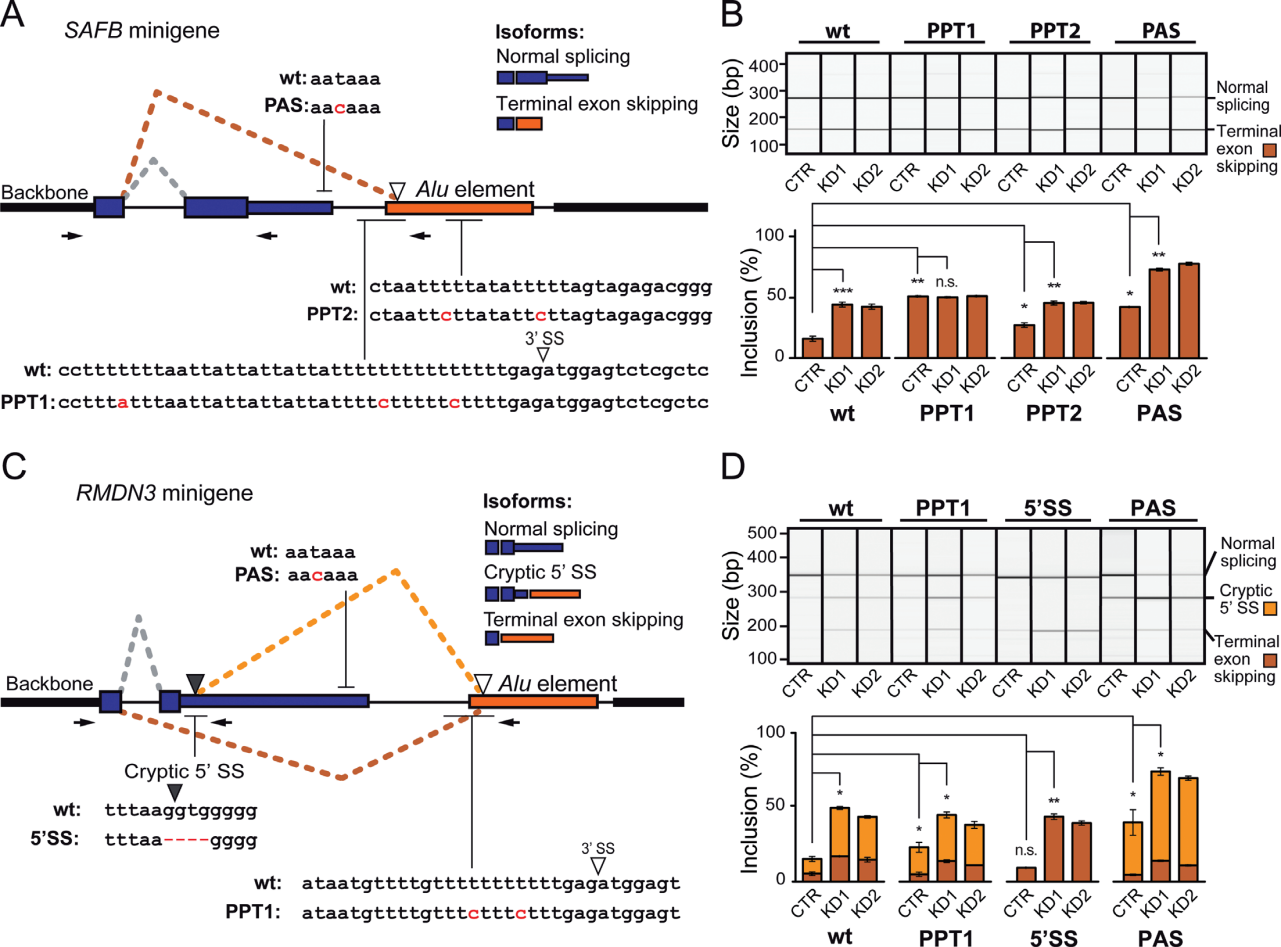
Intergenic *Alu* exonisation is in kinetic competition with preceding splicing and polyadenylation. (**A**) Schematic representation of the *SAFB* minigene. Under normal conditions, intergenic *Alu* exonisation is repressed, the terminal exon is spliced and the transcript is polyadenylated at the genuine polyadenylation site (grey dashed lines). In the *HNRNPC* knockdown, the intergenic *Alu* exon is spliced in favour of the terminal exon (dark orange dashed lines). The white arrowhead marks the 3′ splice site (3′ SS), which is recognized inside the *Alu* element. Depicted above and below are the nucleotide sequences in selected regions of the wild-type construct as well as the introduced mutations (red): polyadenylation signal (PAS), and upstream and downstream polypyrimidine tracts (PPT1 and PPT2, respectively). (**B**) Semiquantitative RT-PCR monitoring the inclusion of the intergenic *Alu* exon in the minigenes with wild-type (wt) or mutated sequences (PPT1, PPT2 and PAS) in *HNRNPC* knockdown (KD1 and KD2) and control HeLa cells (CTR). Top: Gel-like representation of the capillary electrophoresis data showing *Alu* exon inclusion, with the resulting isoforms indicated on the right. Bottom: Quantification of the average *Alu* exon inclusion. Lines indicate relevant comparisons with asterisks representing different levels of significance (*P value < 0.05; **P < 10^−3^; ***P < 10^−4^; n.s., not significant; Student's *t*-test). Error bars represent standard deviation of the mean, n = 3. (**C**) Schematic representation of the *RMDN3* minigene, indicating the splicing pattern under normal conditions (grey) or the two possible scenarios of intergenic *Alu* exonisation upon *HNRNPC* knockdown (cryptic 5′ splice site activation, light orange; terminal exon skipping, dark orange). The black and white arrowheads represent the cryptic 5′ splice site in the terminal exon and the 3′ splice site within the *Alu* element, respectively. Nucleotide sequences and mutations as in (**A**): PAS, upstream polypyrimidine tract (PPT1) and cryptic 5′ splice site (5′SS). (**D**) Semiquantitative RT-PCR monitoring the inclusion of the intergenic *Alu* exon in the *RMDN3* minigene with wild-type (wt) or mutated sequences (PAS, PPT1 and 5′SS) in *HNRNPC* knockdown (KD1 and KD2) and control HeLa cells (CTR). Gel-like representation of capillary electrophoresis (top) and quantification of average *Alu* exon inclusion (bottom) are shown as in (**B**). The two *Alu* exonisation scenarios (cryptic 5′ splice site activation, light orange; terminal exon skipping, dark orange) are indicated on the right.

**Figure 3.**
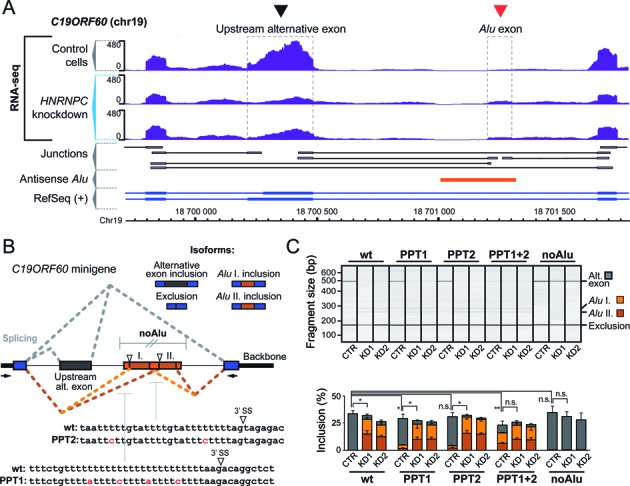
Intronic *Alu* exonisation interferes with the inclusion of an upstream exon. (**A**) Genome browser view of the *C19ORF60* gene (chr19, nt 18 699 714–18 701 786, plus strand) showing the RNA-seq data from control and *HNRNPC* knockdown HeLa cells (labelling as in Figure 1A). The arrowheads mark the hnRNP C-repressed intronic *Alu* exon (red) as well as the upstream alternative exon that is downregulated upon *HNRNPC* knockdown (black). (**B**) Schematic representation of the *C19ORF60* minigene, indicating the splicing pattern under normal conditions when the upstream alternative exon is either included or excluded from the transcript (grey lines), and under *HNRNPC* knockdown conditions when the left and right arm of the intronic *Alu* element exonise and the upstream alternative exon is skipped (light and dark orange lines, respectively). The white arrowheads mark the two 3′ splice sites within the *Alu* element. Indicated below are the nucleotide sequences and mutations for selected regions: upstream polypyrimidine tract (PPT1), downstream polypyrimidine tract (PPT2) and the combination of both (PPT1+2). The region of complete deletion of the *Alu* element is indicated above (noAlu). (**C**) Semiquantitative RT-PCR monitoring the inclusion of the intergenic *Alu* exon in the minigenes with wild-type (wt) or mutated sequences (PPT1, PPT, PPT1+2 and noAlu) in *HNRNPC* knockdown (KD1 and KD2) and control HeLa cells (CTR). Gel-like representation of capillary electrophoresis (top) and quantification of average *Alu* exon inclusion are shown as in Figure 2B. The different detected splicing products (inclusion of the upstream alternative exon, grey; exonisation of the first and second arm of the intronic *Alu* element, light and dark orange, respectively) are indicated on the right.

We used quantitative RT-PCR to measure exon inclusion levels from the minigene constructs in control and *HNRNPC* knockdown HeLa cells (Figures 2B, D and 3C). The RT-PCR templates and reactions were prepared as described above. We used pcDNA3_F oligonucleotide with specific primers for each minigene, listed in Supplementary Table S4. For the visualization of the PCR products and quantification of individual splicing isoforms, we used a QIAxcel capillary gel electrophoresis system (Qiagen).

### 3′ RACE

3′ RACE (rapid amplification of cDNA ends) experiments were performed for the intergenic *Alu* exon in the *RMDN3* gene, similarly as previously described (14). Sequences of oligonucleotides used are listed in Supplementary Table S4. Briefly, 5 μg of total RNA from control and *HNRNPC* knockdown HeLa cells was reverse transcribed using Q_T_ oligonucleotide and RevertAid™ Premium First Strand cDNA Synthesis Kit (Fermentas). The remaining RNA template was destroyed by RNase H. Diluted cDNA template was amplified on the reverse transcription products (30 cycles) using the gene-specific oligonucleotides 1, located in the upstream constitutive exon (RMDN3_GSP1) and Q_o_ oligonucleotide using Phusion High-Fidelity DNA Polymerase (NEB). Diluted amplicon from the first round was additionaly amplified (30 cycles) with the same polymerase and conditions in order to eliminate non-specific products using Q_i_ primer and the gene-specific oligonucleotides 2. These oligonucleotides were specifically designed using information from the RNA-seq data, so that they include the exon-exon junction from the cryptic 5′ splice site to the intergenic *Alu* exon (RMDN3_GSP2a) or the junction from the upstream constitutive exon to the intergenic *Alu* element in the case of terminal exon skipping (RMDN3_GSP2b). The obtained final PCR products were analysed using gel electrophoresis and the products of appropriate sizes were cloned using TOPO TA Cloning Kit (Invitrogen) and sequenced in order to define the 3′ ends of the intergenic *Alu*-derived isoforms.

### Transcript stability measurements

In order to measure transcript isoform stabilities (Figure 5D), cells were treated with 5 μg/ml of the transcription inhibitor actinomycin D at 70% confluence. Cells were collected at different time points and RNA was extracted. Following cDNA synthesis, semiquantitative RT-PCR reactions were carried out in technical triplicates using OneTaq® DNA Polymerase (NEB) and the following conditions: 94°C for 30 s, 28 cycles (*CCDC34*) or 30 cycles (*GALK1*) of [94°C for 20 s, 50°C (*CCDC34*) or 52°C (*GALK1*) for 30 s, 68°C for 30 s] and final extension at 68°C for 5 min. The 2200 TapeStation capillary gel electrophoresis system (Agilent) was used for quantification of the PCR products.

### Intergenic *Alu* exon inclusion in human tissues

The expression of isoforms containing intergenic *Alu* exons was analysed in six different human tissues, including brain, cervix, heart, kidney, liver and testis (FirstChoice total RNA, Life Technologies; Supplementary Figure S7). We used the initially validated hnRNP C-regulated cases (Supplementary Figures S2 and S3). The RT-PCR conditions and quantifications were as described above. The expression was compared to HeLa control and *HNRNPC* knockdown conditions for each analysed case.

### Evolutionary analysis: human and mouse comparison

To identify possible orthology relationships between human genes containing intergenic *Alu* exons and mouse genes, we used the Ensembl Compara tool (15). Among the pairs of orthologues identified, we selected only those, in which the human copy (i) extends beyond the mouse one, (ii) contains at least one *Alu* exon downstream of the terminal exon in mouse and (iii) shows no conservation of exons following the *Alu* exon.

**Figure 4.**
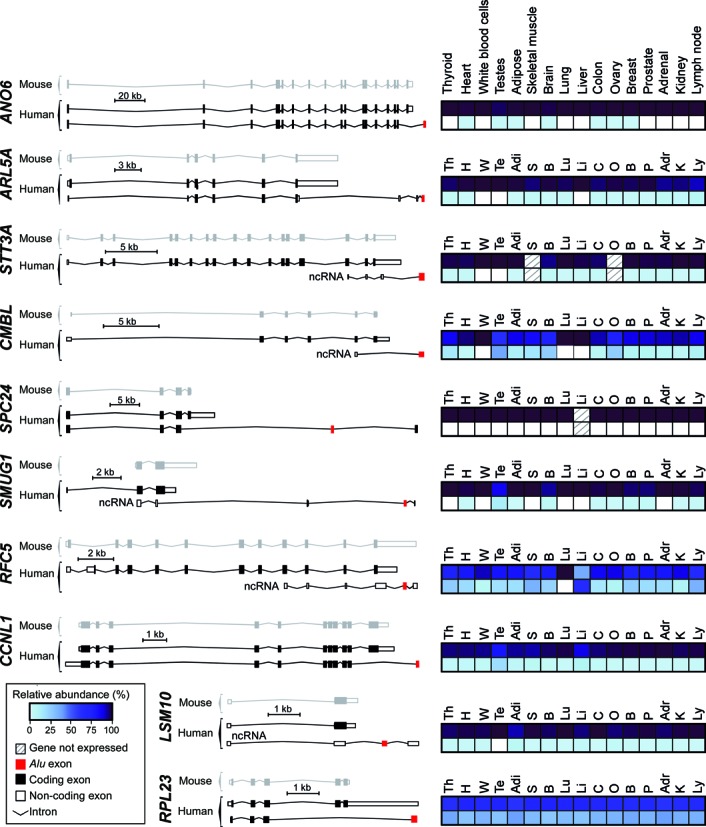
Intergenic *Alu* exonisation facilitated the formation of new transcript 3′ ends during human genome evolution. Comparative genomics identified 10 orthologous gene pairs in the human and mouse genomes, in which the human gene annotation contains an additional transcript isoform with an *Alu* exon (shown in red) downstream of the terminal exon in the mouse orthologue. Coding and non-coding exons are shown as black and white squares, respectively. Only the conserved isoform and the *Alu* exon-containing isoform are shown for each gene. Transcripts are drawn to scale. Quantification of relative isoform expression levels from the Illumina Body Map 2.0 dataset are shown on the right for the *Alu* exon-containing isoforms (bottom) and the sum of all other isoforms (top) in 16 different tissues: adrenal (Adr), thyroid (Th), heart (H), testis (Te), adipose (Adi), skeletal muscle (S), white blood cells (W), brain (B), lung (Lu), liver (Li), colon (C), ovary (O), breast (B), prostate (P), kidney (K), lymph node (Ly). The relative abundance levels were calculated from the fragments per kilobase per million fragments mapped (FPKM) of the *Alu* exon-containing isoforms divided by the sum of FPKM values of all isoforms. Absolute abundance levels and relative levels of the individual isoforms are given in Supplementary Figures S6 and S5, respectively.

For each orthologue pair, we identified the human transcript analogues to the mouse ortholog, and at least one transcript extended by an *Alu* exon and possibly further downstream exons. We used RNA-seq data from Illumina Body Map 2.0 (ArrayExpress accession number E-MTAB-513) (16) to investigate the expression of all human isoforms (Figure 4, Supplementary Figure S5 and S6). Specifically, we downloaded the Fragments Per Kilobase of transcript per Million mapped reads (FPKM) values from DBATE database (17) obtained using Cufflinks (18), which is the normalized expression of individual splicing variants. Supplementary Figure S6 shows the 2-based logarithm of the absolute FPKM values including a pseudocount of 1 [log2(FPKM+1)]. The relative abundance levels were calculated from the FPKM values of the *Alu* exon-containing isoforms divided by the sum of FPKM values of all isoforms. For Figure 4 and Supplementary Figure S6, we grouped the isoforms into *Alu* exon-containing versus all other isoforms. The relative abundance levels of the individual isoforms are given in Supplementary Figure S5 (with all *Alu* exon-containing isoforms labelled in red).

## RESULTS

### The exonisation of downstream intergenic *Alu* elements changes transcript 3′ ends

We previously observed that several hundred *Alu* elements within introns exonise in the absence of the RNA-binding protein heterogeneous nuclear ribonucleoproteins C1/C2 (hnRNP C) (7). When reanalysing the RNA-seq data from *HNRNPC* knockdown HeLa cells, we were surprised to find inclusion of 107 *Alu* exons originating from intergenic regions (called intergenic *Alu* exons, Supplementary Table S1). The intergenic *Alu* exons are linked to the upstream genes. They are located downstream of the last (genuine) polyadenylation site, but are connected via splice junctions to positions within the gene boundaries (Figure 1A), suggesting that they are transcribed as part of the primary transcript. Splicing-mediated inclusion predominates for the majority of intergenic *Alu* exons, as indicated by comparisons of junction-spanning and continuously aligning reads at the *Alu* exon splice sites (Figure 1B). Distances between the intergenic *Alu* elements and the last polyadenylation sites of the corresponding genes range between 41 bp and 21.4 kb (median 3.2 kb; Figure 1C), suggesting that the transcribing RNA polymerase II can progress far beyond the polyadenylation site before cleavage occurs (19).

Inclusion of the intergenic *Alu* exons results from the activation of cryptic splice sites within the *Alu* elements. We observe two scenarios (Figure 1D and E): (i) the complete terminal exon is skipped in favour of the intergenic *Alu* exon (48 cases), or (ii) the intergenic *Alu* exon triggers splicing at a cryptic 5′ splice site within the terminal exon (40 cases; as well as 19 cases in which both scenarios co-occur). The inclusion events result in either complete or partial exchange of the original 3′ UTR sequence. These observations indicate that the intergenic *Alu* exons can distally compete with the preceding splice and/or polyadenylation sites. In addition, 28 of the intergenic *Alu* exons (26.2%) are followed by one or more cryptic exons even further downstream (Supplementary Figure S1), suggesting that intergenic *Alu* exonisation can promote the inclusion of downstream cryptic exons.

To exclude the possibility that the intergenic *Alu* exons arose from artefacts in the RNA-seq data or downstream analysis, we confirmed their inclusion by semiquantitative RT-PCR. Comparing two different *HNRNPC* knockdowns (KD1 and KD2) with control HeLa cells, we validated the increased inclusion of 16 out of 18 intergenic *Alu* exons, including 7 cases of terminal exon skipping, 7 cases of cryptic 5′ splice site activation, and 2 cases, in which both scenarios were observed (Figure 1F, Supplementary Figures S2 and S3, Table S2). Average inclusion levels of the *Alu* exons rise from 12% to 30.7%, with values ranging from 2.4% up to 77.4% in the *HNRNPC* knockdown, confirming that these exons are repressed through hnRNP C (Figure 1F). Many of the intergenic *Alu* exons are detected already in the control cells, indicating that they could have partially lost repression (see below). In summary, we find that the exonisation of intergenic *Alu* elements downstream of genuine polyadenylation sites can create new transcript 3′ ends via terminal exon skipping or cryptic 5′ splice site activation.

### hnRNP C competes with U2AF65 to repress intergenic *Alu* exons

By mapping protein–RNA interactions with individual-nucleotide resolution UV crosslinking and immunoprecipitation (iCLIP), we previously found that hnRNP C competes with the splicing activator U2AF65 to suppress intronic *Alu* exon inclusion (7). In absence of hnRNP C, U2AF65 binds to the continuous uridine tracts (U-tracts) of antisense *Alu* elements, thereby promoting the splicing of hundreds of cryptic *Alu* exons within introns. To test whether the same mechanism applies for the repression of intergenic *Alu* exons, we re-analysed genome-wide iCLIP data for hnRNP C and U2AF65. Consistent with hnRNP C-mediated repression, we observe hnRNP C binding at the U-tracts of intergenic *Alu* exons in control HeLa cells (Supplementary Figure S4A). U2AF65 shows relatively little binding in control cells, but a strong increase upon *HNRNPC* knockdown, indicating competition of the two proteins at these sites (Supplementary Figure S4B). In contrast, U2AF65 binding at preceding exons is not affected by the *HNRNPC* knockdown. Taken together, these results suggest that hnRNP C blocks U2AF65 binding at intergenic *Alu* exons.

To independently validate the competition between hnRNP C and U2AF65 at intergenic *Alu* exons, we constructed a minigene containing the preceding and terminal exon of the *SAFB* gene followed by an intergenic *Alu* element (Figure 2A). As seen for the endogenous transcript, the minigene displays a strong increase in inclusion of the intergenic *Alu* exon upon *HNRNPC* knockdown (Figure 2B). To shift the balance between the two proteins, we introduced mutations in the first U-tract at the 3′ splice site of the *Alu* exon that prevent hnRNP C binding but preserve U2AF65 recognition (PPT1, Figure 2A) (7). This led to strong inclusion of the intergenic *Alu* exon already in the presence of hnRNP C and no further change in the knockdown (Figure 2B). This shows that hnRNP C acts directly at the *Alu* element where it competes with U2AF65. Similar mutations in the second U-tract inside the *Alu* element also led to a slightly elevated *Alu* exon inclusion that further increased upon *HNRNPC* knockdown (PPT2, Figure 2B). Consistent with the model of hnRNP C tetramer binding to two consecutive binding sites (8,20), this suggests that efficient repression requires simultaneous recognition of the two U-tracts through hnRNP C. Taken together, we conclude that hnRNP C prevents U2AF65 from recognizing intergenic *Alu* elements.

### Intergenic *Alu* exonisation competes with splicing and polyadenylation of the preceding gene

Inclusion of intergenic *Alu* exons can happen in conjunction with skipping of the terminal exon, as seen in the example of the *SAFB* minigene. In order to investigate the relationship of intergenic *Alu* exonisation and 3′ end formation, we introduced a point mutation in the genuine polyadenylation signal of the *SAFB* minigene (PAS, Figure 2A). This mutation resulted in strongly elevated *Alu* exonisation in control cells, indicating that loss of polyadenylation and hence stabilization of the downstream transcript region prolongs the window of opportunity for inclusion of the *Alu* exon. For this mutation, *HNRNPC* knockdown still led to an increase comparable to the wild-type construct, showing that shifting the balance between exonisation and polyadenylation does not influence hnRNP C-mediated repression of the *Alu* exon (Figure 2B).

Our results demonstrate that intergenic *Alu* exonisation can compete with both splicing and polyadenylation of the preceding terminal exon (21). This suggests that each of the three processing steps has a certain probability to happen first. Depending on this initial choice, different outcomes are possible: (i) initial splicing of the terminal exon has to be followed by normal polyadenylation, (ii) immediate polyadenylation precludes *Alu* exonisation and hence promotes normal splicing of the terminal exon, and (iii) initial splicing of the intergenic *Alu* exon dictates skipping of the terminal exon together with the genuine polyadenylation site. Consequently, either slowing down or impairing polyadenylation, as in the case of the PAS mutation, or modulating the splicing kinetics of the intergenic *Alu* exon, as in the case of the *HNRNPC* knockdown and the PPT mutations, can shift the balance between the three processing steps.

To dissect the interplay of splicing and polyadenylation, we constructed a second minigene from the *RMDN3* gene. Here, inclusion of an intergenic *Alu* exon can trigger either skipping of the terminal exon or activation of a cryptic 5′ splice site (Figures 1A and 2C). Polyadenylation then happens at a new site downstream of the *Alu* exon, which we experimentally validated using 3′ RACE (Figure 1A). As seen for the *SAFB* minigene, *Alu* exonisation can be further enhanced by *HNRNPC* knockdown, by mutations relieving hnRNP C repression or by mutating the polyadenylation site (PPT1, PAS; Figure 2C and D). In all cases, activation of the cryptic 5′ splice site seems the favoured outcome. Surprisingly, mutating this splice site does not change overall exonisation levels, but completely shifts the outcome to skipping of the terminal exon (5′SS; Figure 2C and D). These observations support the idea that the exonisation decision is made primarily at the *Alu* exon, which is in kinetic competition both with polyadenylation and splicing of the preceding terminal exon. In this interplay, the choice of the partnering 5′ splice site seems to be secondary, at least in this case, with possibly the closest suitable 5′ splice site being used by default. In summary, we conclude that splicing and polyadenylation of the terminal exon can be in kinetic competition with splicing of the *Alu* exons in the intergenic region.

### Intronic *Alu* exonisation impairs the inclusion of upstream alternative exons

Since intergenic *Alu* exonisation is in kinetic competition with preceding splicing and polyadenylation, we assessed whether *Alu* exons within introns could similarly compete with splicing of internal upstream exons. One such example can be seen in the *C19ORF60* gene, which shows strongly decreased inclusion of the upstream alternative exon (2.2-fold, adjusted *P*-value < 0.01; Figure 3A). To investigate whether this downregulation is a direct consequence of *Alu* exonisation, we generated a minigene containing the upstream alternative exon and the intronic *Alu* element flanked by two constitutive exons (Figure 3B). As seen for the endogenous transcript, knockdown of *HNRNPC* results in exonisation from both arms of the *Alu* element in conjunction with almost complete skipping of the upstream alternative exon (wt, Figure 3C). Mutations in both U-tracts of the *Alu* element, that relieve hnRNP C repression, lead to strong *Alu* exon inclusion that is accompanied by alternative exon skipping already in control cells (PPT1, PPT2, PPT1+2; Figure 3C). Inversely, completely removing the *Alu* element abolishes any regulation of the upstream alternative exon in the *HNRNPC* knockdown (noAlu; Figure 3C). These observations are consistent with the model that emerging *Alu* exons kinetically compete with processing of the upstream alternative exons, with the same phenomenon occurring both inside and downstream of genes.

### Intergenic *Alu* exonisation facilitated the evolution of transcript 3′ ends in human

We started out with the initial observation that the intergenic *Alu* elements show exonisation in the *HNRNPC* knockdown. However, 75% of these exons show considerable inclusion already in control HeLa cells (Figure 1F). This prompted us to investigate whether the intergenic *Alu* exons might form parts of functional transcripts or represent intermediate states of generating new transcript 3′ ends. To search for evidence of such events during evolution, we compared human genes with their orthologues in the mouse genome, which is devoid of the primate-specific *Alu* elements. Intriguingly, we found 10 human genes with annotated isoforms that carry an *Alu* exon downstream of the terminal exon in the mouse orthologue. These include the *ANO6* gene, in which the complete 3′ UTR as conserved in the mouse gene is exchanged in the *Alu* exon-containing isoform (Figure 4). The *Alu* exons can also form part of the protein-coding region, as seen in the case of the cyclin L1 gene (*CCNL1*; Figure 4). These instances demonstrate that the 3′ UTR as well as parts of the encoded protein can be exchanged through inclusion of an intergenic *Alu* exon. In summary, we find that the human genome contains several annotated transcript isoforms with 3′ ends that most likely originated from intergenic *Alu* exonisation events.

### *Alu*-derived isoforms are expressed in a tissue-specific fashion

**Figure 5.**
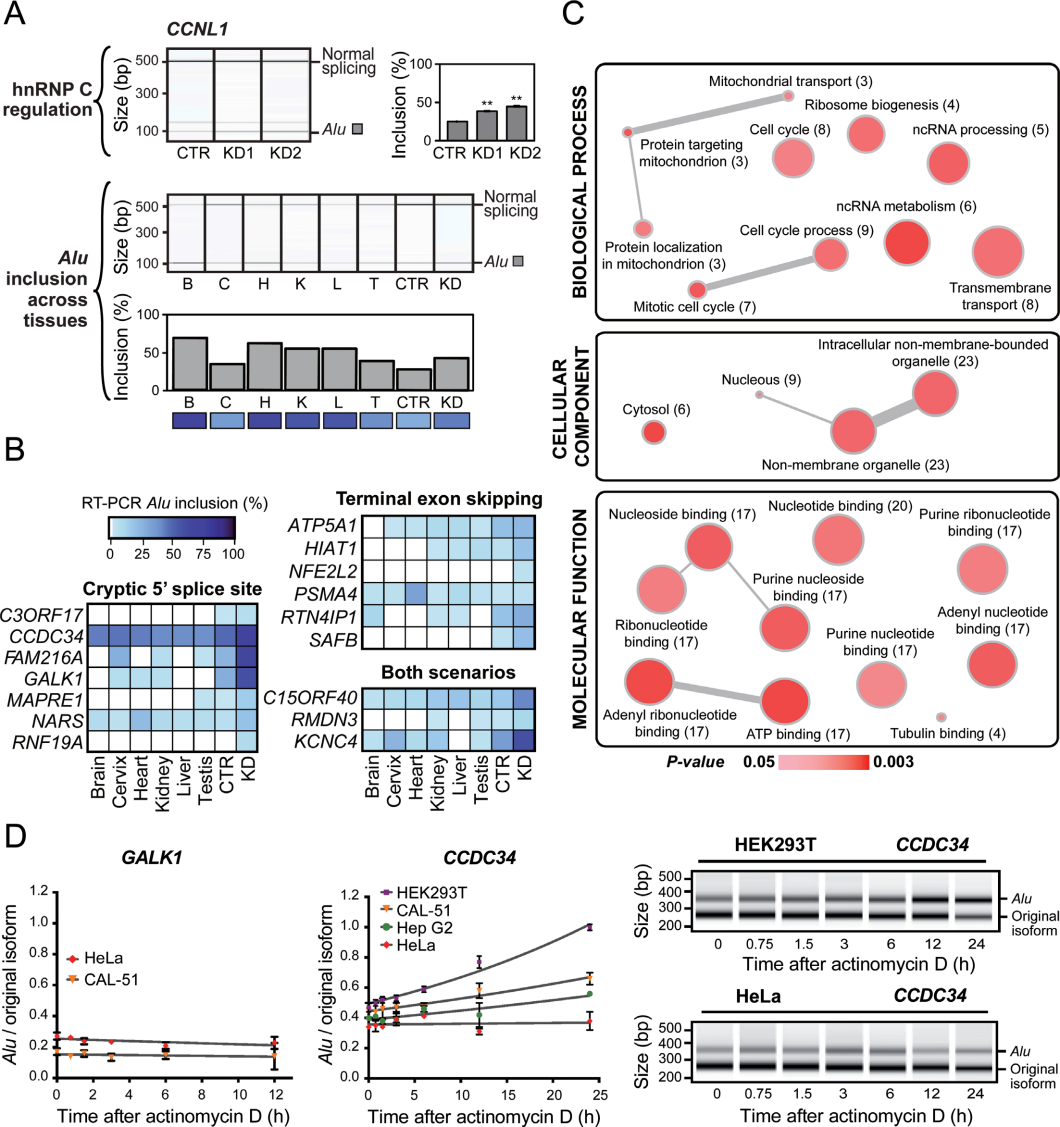
Intergenic *Alu* exons show tissue-specific expression and are enriched in regulatory genes. (**A**) Semiquantitative RT-PCR monitoring the annotated *Alu* exon-containing isoform of the cyclin L1 gene (*CCNL1*) that is regulated by hnRNP C (top; KD1 and KD2 are shown). *Alu* exon inclusion was measured across six different human tissues (bottom panels): brain (B), cervix (C), heart (H), kidney (K), liver (L), testis (T), as well as control (CTR) and *HNRNPC* knockdown (KD = KD1) HeLa cells. Shown are gel-like representations of capillary electrophoresis and quantifications of average *Alu* exon inclusion. A heatmap-type representation is given below to facilitate comparison with (**B**). For HeLa cell data (top panels), asterisks represent the level of significance (**P < 10^−3^; Student's t-test), and error bars represent standard deviation of the mean, n = 3. (**B**) *Alu* inclusion levels of 16 intergenic *Alu* exons across 6 different human tissues: brain (B), cervix (C), heart (H), liver (L), kidney (K) and testis (T), measured by semiquantitative RT-PCR, including the measures of control (CTR) and *HNRNPC* knockdown (KD = KD1) HeLa cells. (**C**) Gene Ontology (GO) analysis of the 107 genes with intergenic *Alu* exonisation events upon *HNRNPC* knockdown, using the DAVID Gene Ontology Tool and visualized using ReviGO. Node colour indicates the *P* value (threshold: *P* value < 0.05), and node size indicates the frequency of the GO term in the GOA database. Each gene is mapped only to the most specific terms that are applicable to it (in each ontology). The number of genes with intergenic *Alu* exonisation in each category is given in brackets. Highly similar GO terms are linked by edges in the graph, with the edge width depicting the degree of similarity. (**D**) Transcript stability measurements upon transcriptional inhibition with actinomycin D. Semiquantitative RT-PCR monitoring the relative abundance of the *Alu*-derived isoform over time across a panel of four different cell lines (HEK293T, CAL-51, Hep G2 and HeLa). Left, quantification of average abundance ratios of the *Alu*-derived versus the original isoform. Trend lines from exponential growth equations are shown for each cell line. Error bars represent standard deviation of the mean, n = 3, technical replicates. Right, gel-like representations of capillary electrophoresis of the *Alu*-derived and original isoforms in HEK293T and HeLa cells.

Since 3′ UTRs serve as hubs for posttranscriptional regulation, they often confer tissue-specific expression. To test whether the annotated *Alu*-derived isoforms show tissue specificity, we examined their relative abundance in 16 primary human tissues using the Illumina Body Map 2.0 data set (16). For 9 out of 10 genes, we observe tissue-specific variations in expression of the *Alu* exon-containing isoforms (Figure 4, Supplementary Figures S5 and S6). The *Alu*-derived isoforms represent the minor isoforms in most cases, but show significant expression in selected tissues. In the case of the *RFC5* gene, the *Alu*-derived isoform is the single most abundant isoform in several tissues (skeletal muscle, liver and lymph node; Supplementary Figure S5); e.g. it accounts for more than 75% of the gene's total expression in liver, while it shows no expression at all in lung (Figure 4). For *CCNL1*, we independently confirmed varying inclusion levels across tissues using semiquantitative RT-PCR (Figure 5A).

The prevalence of tissue-specific expression among the annotated *Alu*-derived isoforms prompted us to re-evaluate the inclusion of the hnRNP C-repressed intergenic *Alu* exons. Using semiquantitative RT-PCR, we measured the inclusion levels of 16 intergenic *Alu* exons across six different human tissues: brain, cervix, heart, liver, kidney and testis. We find more than 10% inclusion in at least one tissue for 7 out of 16 (44%) tested intergenic *Alu* exons, including three with terminal exon skipping, three with activation of a cryptic 5′ splice site and one with both scenarios (Figure 5B and Supplementary Figure S7). Inclusion can rise up to 45% as in the case of the *CCDC34* gene (Figure 5B and Supplementary Figure S7). Whereas some of the exons show homogeneous inclusion, others display strong tissue specificity as seen in the *KCNC4*, *PSMA4* and *FAM216A* genes (Figure 5B and Supplementary Figure S7), suggesting an additional layer of tissue-dependent regulation.

Several molecular mechanisms could explain the tissue-specific expression of the *Alu*-derived isoforms: in addition to tissue-specific splicing and polyadenylation patterns, it is conceivable that the newly introduced 3′ UTRs are subject to different modes of posttranscriptional regulation, e.g. at the level of transcript stability. To address this, we measured the relative stability of the *Alu*-derived and original isoforms of the *CCDC34* and *GALK1* genes upon treatment with the transcriptional inhibitor actinomycin D across a panel of four different cell lines (HEK293T, CAL-51, Hep G2 and HeLa; Figure 5D). For *GALK1*, we detect expression of the *Alu*-derived isoform in steady state only in HeLa and CAL-51 cells, and the two transcript isoforms show equal stability in both cell lines. Thus, the expression differences of the *Alu*-derived isoform between these two cell lines most likely result from changes in alternative splicing and/or polyadenylation. In contrast, the *Alu*-derived isoform of *CCDC34* is considerably more stable than the original isoform in HEK293T cells, while they exhibit equal stability in HeLa cells. Notably, the relative stability in the four cell lines matches the respective relative abundance of the *Alu*-derived *CCDC34* isoform in steady state (t = 0), which is highest in HEK293T cells and lowest in HeLa cells. Thus, the stability differences can vary between cell lines, which explains how the *Alu*-derived isoforms could mediate cell- or tissue-specific gene expression.

In conclusion, we find that some *Alu* exon-containing isoforms are expressed in human in a tissue-specific manner. Intriguingly, we observe considerable tissue-specific regulation also for several of the intergenic *Alu* exons that we initially identified in the *HNRNPC* knockdown, suggesting that these isoforms constitute *bona fide* alternative isoforms of the respective genes. That these isoforms are not present in current genome annotations might be due to the repetitive nature of the included *Alu* element fragments, which impairs their unambiguous assignment.

### Intergenic *Alu* exonisation is enriched in genes that function in DNA or RNA metabolism

We next wanted to explore the possible functional consequences of intergenic *Alu* exonisation. While we initially detected the intergenic *Alu* exons in the *HNRNPC* knockdown, almost half of the tested examples showed considerable inclusion across tissues, indicating that the *Alu*-derived isoforms could represent *bona fide* isoforms that are functionally relevant. We therefore decided to use the DAVID GO tool (11) on the full set of 107 genes in which we detect intergenic *Alu* exons. We find significant enrichment in genes which are important for cell cycle and non-coding RNA (ncRNA)-related processes, among others (Figure 5C, Supplementary Table S3). Furthermore, significantly enriched molecular functions are connected to DNA/RNA-related processes, such as ribonucleotide and nucleoside binding (Figure 5C, Supplementary Table S3). Consistent with these observations, previous results indicate that mobile elements like *Alu* elements tend to insert in the vicinity of genes that are important for DNA-dependent processes, most likely due to the opening of the chromatin during germline transposition (22). It is therefore conceivable that these genes are particularly prone to undergo *Alu* exonisation-mediated transcript modifications.

## DISCUSSION

**Figure 6.**
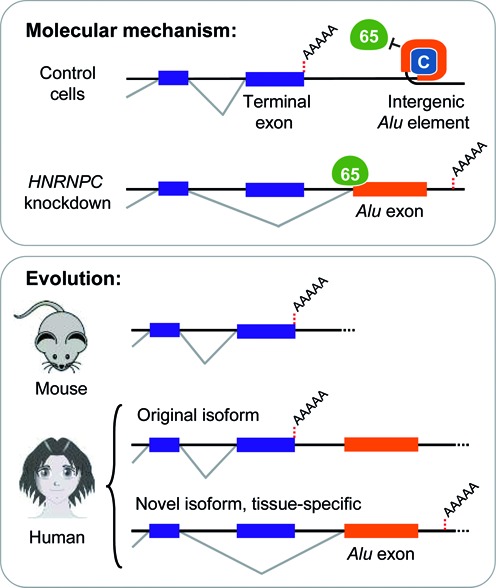
Intergenic *Alu* exonisation facilitated the formation of alternative transcript 3′ ends during human genome evolution. Shown is a schematic model of the hnRNP C-mediated regulation of intergenic *Alu* exonisation and its impact on the formation of new transcript 3′ ends during human genome evolution. Upper panel: Under normal conditions, recognition of the splice sites and exonisation of the intergenic *Alu* element is repressed by hnRNP C, which prevents U2AF65 from binding to the continuous U-tracts of the *Alu* element. Under these conditions, the terminal exon is correctly spliced and polyadenylation occurs at the genuine polyadenylation site. In the absence of hnRNP C, the splice sites of the intergenic *Alu* element are recognized by the splicing machinery that promotes *Alu* exonisation. As a consequence, the terminal exon is either skipped or spliced to the exonised intergenic *Alu* element through activation of a cryptic 5′ splice site inside the terminal exon (not shown). In both cases, the transcript is polyadenylated at a new downstream polyadenylation site (dashed red line). Lower panel: Schematic comparison of orthologous genes in the human genome and the *Alu* element-free mouse genome. In the human orthologue, transcripts either end with the conserved terminal exon (top) or contain an *Alu* exon downstream of last common exon (bottom).

In this study, we use genome-wide assays, comparative genomics and minigene experiments to show that intergenic *Alu* elements can compete with genuine polyadenylation sites to introduce new transcript 3′ ends (Figure 6). At the mechanistic level, we observe the kinetic competition of *Alu* exonisation with splicing and polyadenylation of the preceding terminal exon. This means that the polyadenylation machinery must race to cleave at the canonical polyadenylation site before splicing at the *Alu* exon occurs. However, we find that polyadenylation is not fast enough to match the increased efficiency of intergenic *Alu* exon splicing upon *HNRNPC* knockdown. Resulting inclusion of intergenic *Alu* exons can change the composition of 3′ UTRs, modifying posttranscriptional regulation of the newly emerging isoforms, e.g. at the level of transcript stability. Intriguingly, we provide evidence that intergenic *Alu* exonisation served as a mechanism to evolve new tissue-specific transcript isoforms. We thus present a novel mechanism by which transposable elements within intergenic regions can function in driving the evolution of 3′ UTRs in the human genome.

### The role of *Alu* elements in polyadenylation

Previous studies established that TEs significantly contributed to the repertoire of polyadenylation sites in the human transcriptome, mostly by introducing new polyadenylation signals that are embedded within the TE sequence. For instance, the polyA stretches in sense *Alu* elements have a high propensity to mutate into the canonical polyadenylation signal AAUAAA, as seen in hundreds of human transcripts (23,24). To a lesser extent, CG-rich regions of antisense *Alu* elements developed into auxiliary elements that activate upstream polyadenylation sites in close vicinity (23). In a more indirect mode of action, intronic *Alu* elements have also been described as ‘speed bumps’ that slow down the elongating polymerase and thereby enhance the usage of upstream splice and cryptic polyadenylation sites (25). Here, we present a novel mechanism by which *Alu* elements can diversify the polyadenylation pattern of human genes. We find that *Alu* elements can act from an intergenic position to interfere with 3′ end processing. The splicing-mediated exonisation of these *Alu* elements, that are often several kb downstream of the genuine polyadenylation site, introduces new terminal exons that outcompete splicing and polyadenylation of the genes’ original terminal exons (see below). Cleavage and polyadenylation then occurs at a new polyadenylation site downstream of the intergenic *Alu* element, most likely the first suitable PAS that is encountered by the transcribing RNA polymerase II.

### The kinetic competition of *Alu* exonisation, splicing and polyadenylation

Most mRNA processing takes place in a highly coordinated manner, with individual processing steps affecting each other. For instance, the splicing reaction at one exon can exert an influence on neighbouring splice sites. In a special constellation of splice-site competition, decoy 3′ splice sites within introns engage with 5′ splice sites of upstream exons, thereby preventing their usage by the spliceosome (26–28). Relatedly, it was shown that introducing an exonisation-competent *Alu* element can reduce the inclusion of an upstream constitutive exon (28,29). Using minigene experiments, we demonstrate that an intronic *Alu* exon that is normally repressed by hnRNP C, can impair the inclusion of an upstream alternative exon. The *Alu* exon is spliced in a mutually exclusive way, indicating that it is in direct competition with the upstream alternative exon. This competition most likely emanates from the cryptic 3′ splice site in the *Alu* exon, since mutations that enhance recognition of this 3′ splice site are sufficient to reinforce skipping of the upstream alternative exon.

In addition to the influence on neighbouring splice sites, there are intimate links between splicing and 3′ end processing. In this interplay, both cooperation and competition have been observed. For instance, the efficiency of cleavage and polyadenylation is increased by terminal exon definition, and several components of the spliceosome exhibit direct molecular contacts with 3′ end processing factors (30,31). On the other hand, U1 snRNP can suppress cleavage and polyadenylation at nearby sites in a process called telescripting, thereby protecting the transcriptome against premature 3′ processing at cryptic PASs within introns (32,33). Here, we find that the exonisation of intergenic *Alu* elements impairs 3′ end processing at the genuine polyadenylation sites of genes. Splicing of the *Alu* exon is accompanied either by complete skipping of the terminal exon or by activation of a cryptic 5′ splice site within the terminal exon. Our minigene experiments strongly support the hypothesis that this change in 3′ end processing starts from the *Alu* exon, since mutations relieving repression of this exon are sufficient to lower the utilisation of the genuine polyadenylation site. This effect could result from two possible scenarios: either splicing of the *Alu* exon is in kinetic competition with polyadenylation, or it actively represses the upstream polyadenylation site. Our data are in favour of the kinetic competition model (21), since mutating the genuine polyadenylation site enhances *Alu* exonisation, most likely by extending the window of opportunity for splicing at the intronic *Alu* element. This would argue that PAS recognition occurs before splicing, as it was previously described in the case of a cryptic last exon within an intron (32). Notably, a substantial number of intergenic *Alu* exonisation events involve the activation of a cryptic 5′ splice site within the terminal exon. Mutating the cryptic 5′ splice site does not restore polyadenylation efficiency in our minigene experiments, arguing against a telescripting-like role of U1 snRNP in suppressing the genuine polyadenylation site, most likely because it is out of the range of U1 snRNP suppression of 500–1000 nt (32). Taken together, our data suggest that intergenic *Alu* exonisation is in kinetic competition with splicing and 3′ end processing of the preceding terminal exon.

### The contribution of *Alu* exonisation to 3′ UTR evolution

The 3′ UTRs are major regulatory loci involved in numerous processes, such as the stability, subcellular localisation and translation of mRNAs. Human genes commonly give rise to more than one transcript isoform with different 3′ UTR sequences, allowing for differential binding of regulatory proteins and microRNAs (miRNAs), among others (1). The relative expression of different 3′ UTR isoforms is often tissue-restricted and varies across development stages, opening the possibility to exert tissue-specific fine-tuning also for genes that are ubiquitously expressed (34,35). In line with their contribution to transcriptome diversity, 3′ UTRs evolutionary expanded with increasing organismic complexity, culminating in an average 3′ UTR length in human that is more than twice as long as in other mammals, including mice (34,36). It is also well accepted that an increasing number of disease-causing mutations are mapped to 3′ UTRs, often causing defects in protein translation (37).

Here, we present the exonisation of *Alu* elements from intergenic regions as a novel mechanism to reshape transcript 3′ ends and to introduce new terminal exons. In a substantial number of cases, the *Alu* exon triggers the inclusion of additional cryptic exons, thereby further extending the newly inserted 3′ UTR sequence. Intriguingly, although recognition of the intergenic *Alu* exons is generally attenuated by hnRNP C, many of them are detectable already under control conditions and/or show substantial variability across human tissues. This observation suggests that they might represent intermediate states of newly emerging transcript 3′ ends or even form parts of functional transcripts.

In addition to tissue-specific effects on alternative splicing of the *Alu* exons, the newly introduced 3′ UTRs might serve as targets of further posttranscriptional regulatory processes. The *Alu* element fragments, that are incorporated into the mature transcripts, have a high propensity to form secondary structures, to contribute AU-rich elements (AREs) (38) and to act as miRNA landing sites (39). Moreover, a recent study evaluated RBP binding to repetitive elements and found that various RNA-binding proteins (RBPs) specifically recognize distinct sites within *Alu* elements (39,40). Together, these observations suggest that the *Alu*-derived isoforms could underlie different posttranscriptional targeting. Indeed, we show that the *Alu*-containing isoform can deviate from the original isoform in terms of stability, and that this differential stability can vary considerably between cell lines. It is conceivable that the *Alu*-derived isoform could evade posttranscriptional processes targeting the original isoform by replacing the target region in its 3′ UTR. Consistently, we find that the original 3′ UTRs contain a multitude of predicted binding sites for various miRNA and RBPs (41,42) that are lost in the *Alu*-derived isoforms (data not shown). Vice versa, the newly generated 3′ UTRs could harbour new miRNA and RBP binding sites, as suggested by previous studies (39,40). On the other side of the coin, we find that, e.g. the newly generated 3′ UTR of *NOMO3* harbours the *miR-3179–2* gene (Supplementary Figure S8), suggesting that processing of the *Alu*-derived isoform could increase *miR-3179-2* levels and thereby affect the expression of further genes. Despite *Alu* exonisation, expression of the original transcript isoform is never completely lost, suggesting that alternative splicing of the intergenic *Alu* exons enables evolutionary testing of the novel 3′ UTRs, while simultaneously preserving the functionality of the original isoform. Importantly, comparison with the mouse genome revealed several genes in the human genome that show evidence of transcript 3′ end evolution via intergenic *Alu* exonisation.

In essence, our findings emphasize the dynamic nature of the gene organization in the human genome that underwent extensive changes during evolution. Since *Alu* elements are highly repetitive, *Alu*-derived isoforms can create major challenges for genome annotation. Moreover, if these isoforms are overlooked, it easily distorts transcript quantifications from genome-wide assays, such as RNA-seq data. The impairment is likely to be profound, since we find that some of the intergenic *Alu* exons display almost 50% inclusion in certain tissues. In the future, our findings might also help to interpret disease-associated SNPs within intergenic regions that are studied to date mostly in the context of transcriptional enhancer regions. Our results thus provide a framework for studies of disease-associated intergenic sequence variants that may affect the tissue-specific processing and expression of preceding genes.

## Supplementary Material

SUPPLEMENTARY DATA
