# Supplementary material for: Intergenic Alu exonisation facilitates the evolution of tissue-specific transcript ends
Source: Nucleic Acids Res. 2015 Sep 22;43(21):10492–505. doi: 10.1093/nar/gkv956 (PMC4666398; doi:10.1093/nar/gkv956)
Supplement: SUPPLEMENTARY DATA [file supp_43_21_10492__index.html]

Intergenic Alu exonisation facilitates the evolution of tissue-specific transcript ends — SUPPLEMENTARY DATA 

# Intergenic *Alu* exonisation facilitates the evolution of tissue-specific transcript ends

## SUPPLEMENTARY DATA

- SUPPLEMENTARY DATA
