# Supplementary material for: Intergenic Alu exonisation facilitates the evolution of tissue-specific transcript ends
Source: Nucleic Acids Res. 2015 Sep 22;43(21):10492–505. doi: 10.1093/nar/gkv956 (PMC4666398; doi:10.1093/nar/gkv956)
Supplement: SUPPLEMENTARY DATA [file supp_gkv956_nar-00374-z-2015-File008.pdf]

Intergenic *Alu* exonisation facilitates the evolution of tissue-specific transcript ends

SUPPLEMENTARY MATERIAL

Mojca Tajnik[1,2,9], Alessandra Vigilante[3,4,9], Simon Braun[5], Heike Hänel[5], Nicholas M. Luscombe[3,4,6], Jernej Ule[1,7], Kathi Zarnack[4,8,*] and Julian König[1,5,7,*]

[1] MRC Laboratory of Molecular Biology, Francis Crick Avenue, Cambridge Biomedical Campus, Cambridge CB2 0QH, UK. [2] International Centre for Genetic Engineering and Biotechnology, Padriciano 99, 34149 Trieste, Italy. [3] UCL Genetics Institute, Department of Genetics, Evolution & Environment, University College London, Gower Street, London WC1E 6BT, UK. [4] Cancer Research UK London Research Institute, 44 Lincoln's Inn Fields, London WC2A 3LY, UK. [5] Institute of Molecular Biology (IMB) gGmbH, Ackermannweg 4, 55128 Mainz, Germany. [6] Okinawa Institute of Science & Technology, 1919-1 Tancha, Onna-son, Kunigami-gun, Okinawa 904-0495, Japan. [7] Department of Molecular Neuroscience, UCL Institute of Neurology, Queen Square, London WC1N 3BG, UK. [8] Buchmann Institute for Molecular Life Sciences (BMLS), Max-von-Laue-Str. 15, 60438 Frankfurt, Germany

[9] These authors contributed equally to this work.

* Correspondence: kathi.zarnack@bmls.de (K.Z.), j.koenig@imb-mainz.de (J.K.)

Figure S1

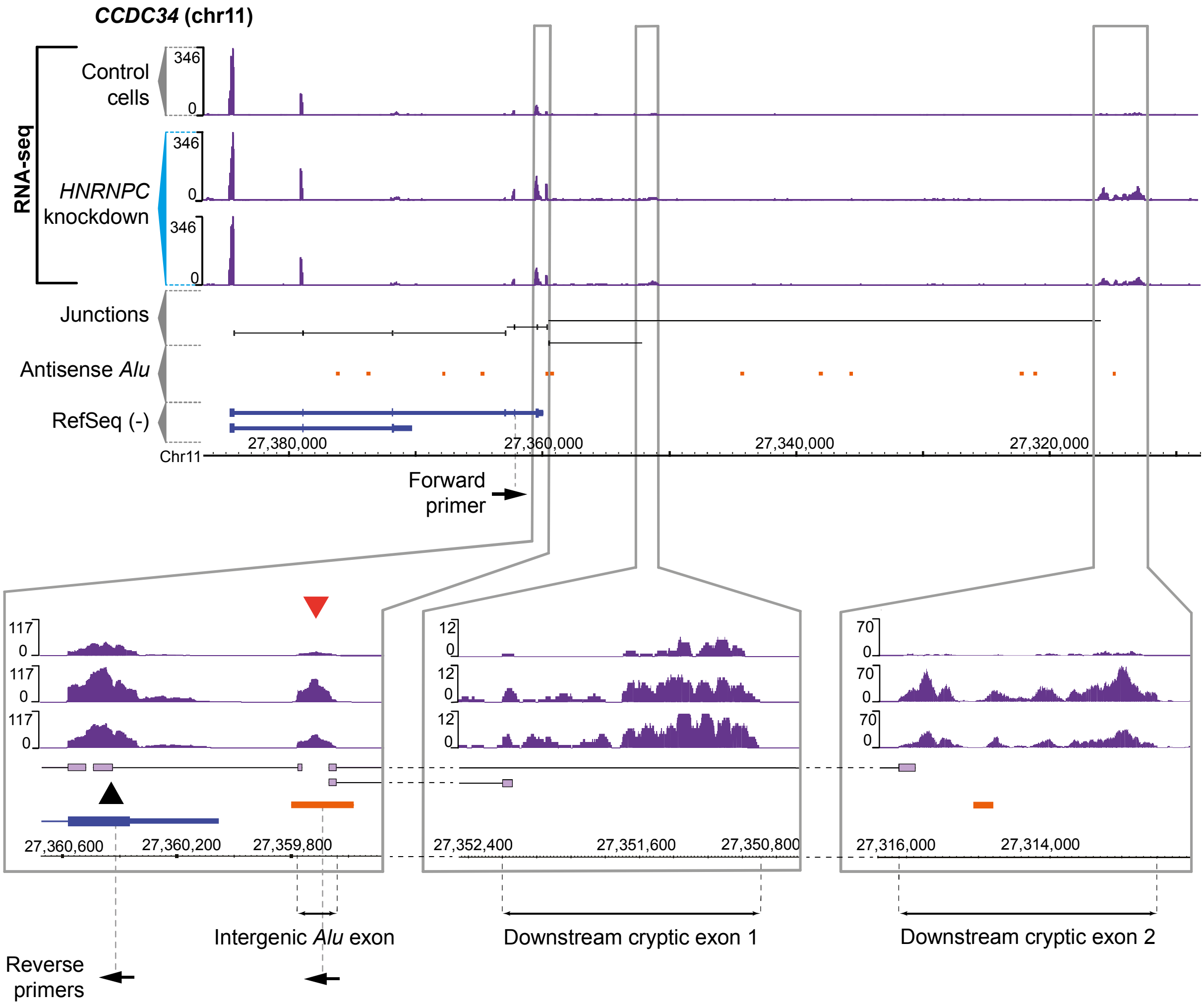

Figure S1 Intergenic *Alu* exonisation promotes the inclusion of downstream cryptic exons.

Genome browser view of the *CCDC34* gene (chr11, nt 27,308,294-27,386,708, minus strand) presenting the RNA-seq data from control and *HNRNPC* knockdown HeLa cells (labeling as in Figure 1A). Three zoom-in views are shown below: the exonisation of the intergenic *Alu* element (red arrowhead) in absence of hnRNP C via activation of a cryptic 5' splice site (black arrowhead) within the terminal exon (chr11, nt 27,359,506-27,360,648; left panel), followed by the two different downstream cryptic exons (chr11, nt 27,350,662-27,352,654, middle panel; chr11, nt 27,312,044-27,316,403; right panel). The location of the RT-PCR primers are depicted below (not to scale).

Figure S2

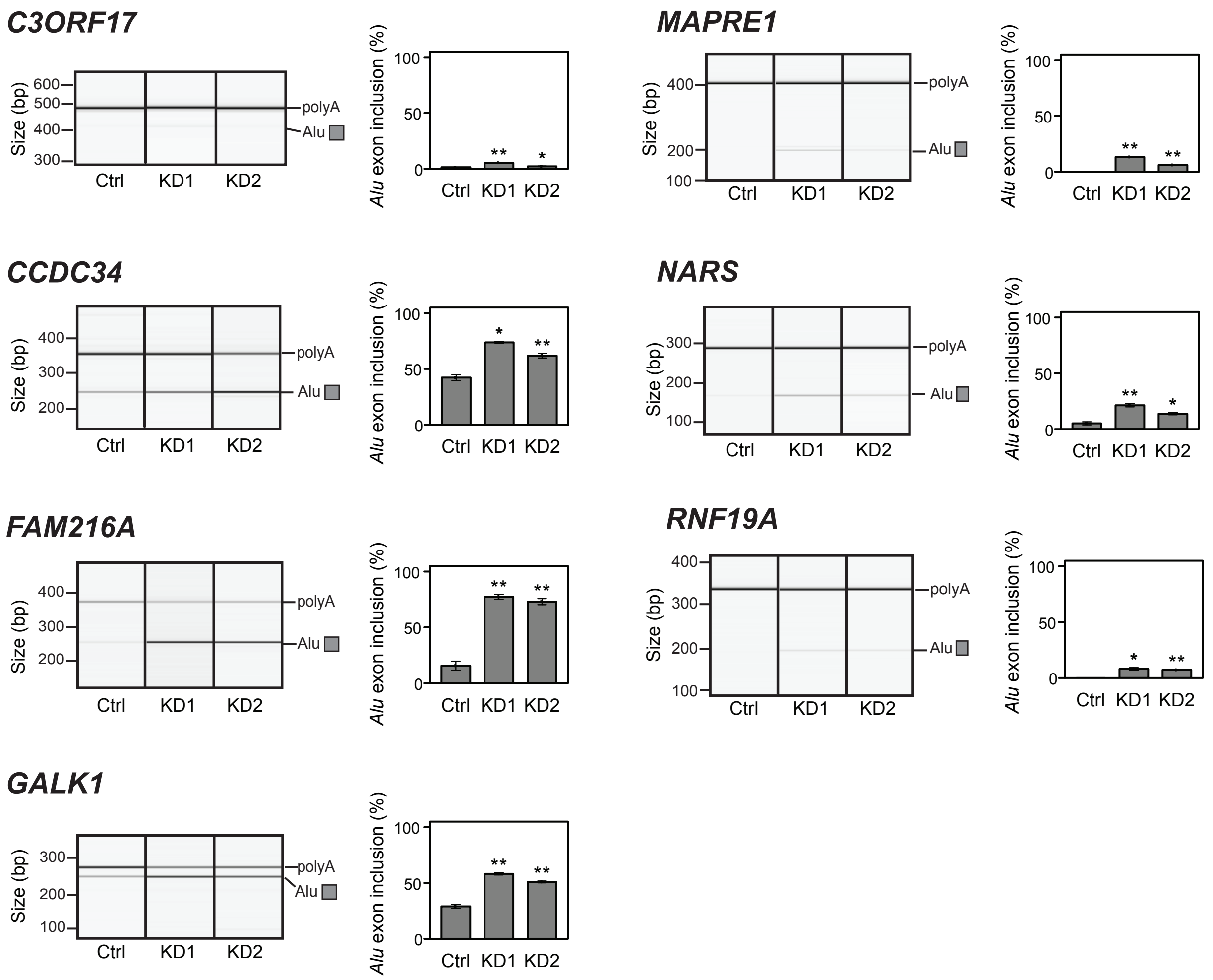

Figure S2 Quantification of intergenic *Alu* exonisation via cryptic 5' splice site activation.

Semiquantitative RT-PCR monitoring intergenic *Alu* exonisation employing activation of a cryptic 5' splice site within the terminal exon. *Alu* exon inclusion was measured in control (Ctrl) and *HNRNPC* knockdown (KD1 and KD2) HeLa cells. Shown are gel-like representations of capillary electrophoresis (left) and quantification of average *Alu* exon inclusion levels (right) for 7 validated cases of cryptic 5' splice site activation (out of 8 cases tested). Gene names are given above (Table S2). Asterisks represent different levels of significance when compared to control conditions (*p value < 0.05; **$p < 10^{-3}$; ***$p < 10^{-4}$; Student's t-test). Error bars represent standard deviation of the mean, n=3.

Figure S3

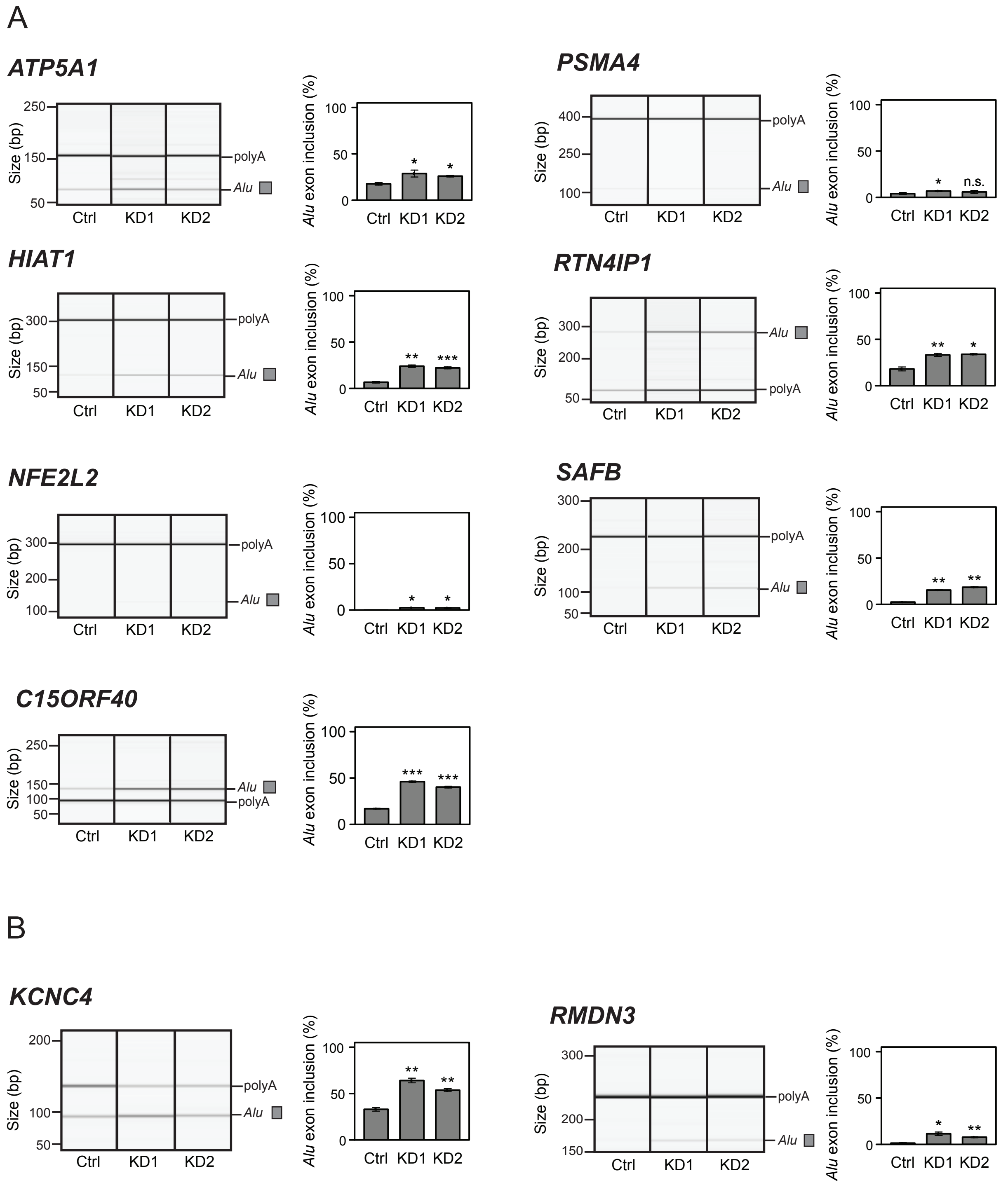

Figure S3 Quantification of intergenic *Alu* exonisation via terminal exon skipping or both scenarios.

Semiquantitative RT-PCR monitoring intergenic *Alu* exonisation employing either (A) terminal exon skipping, or (B) both scenarios co-occurring. *Alu* exon inclusion was measured in control (Ctrl) and *HNRNPC* knockdown (KD1 and KD2) HeLa cells. Measurements are shown for 7 validated cases of terminal exon skipping (out of 7 tested) and 2 validated cases in which both cryptic 5' splice site activation and terminal exon skipping co-occur (out of 3 tested). polyA indicates normal splicing, i.e. generation of the original isoform. Note that in the case of both scenarios co-occurring in our RNA-seq data, we commonly detect only one isoform in the RT-PCR reactions, possibly because the minor isoform is below detection limits. Presentation as in Figure S2.

Figure S4

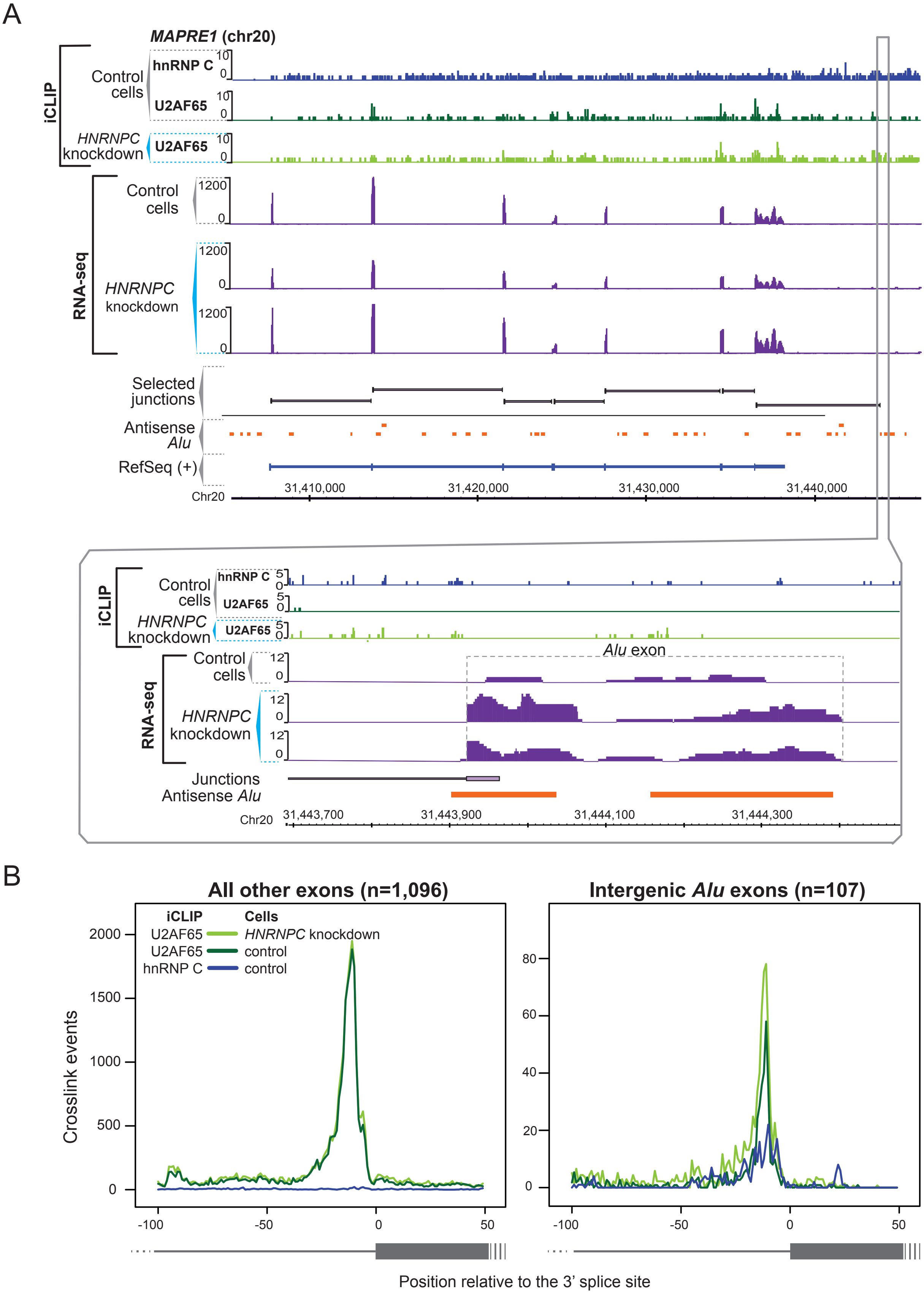

Figure S4 hnRNP C competes with U2AF65 for binding at intergenic *Alu* exons.

(A) Genome browser view of *MAPRE1* gene (chr20, nt 31,405,404-31,446,272, plus strand) presenting iCLIP data of hnRNP C binding (blue) and U2AF65 binding from control (dark green) and *HNRNPC* knockdown (light green) conditions as well as RNA-seq data (purple) from control and *HNRNPC* knockdown (KD1 and KD2) HeLa cells. A zoom-in view of the region including the intergenic *Alu* exon is show below (chr20, nt 31,443,694-31,444,479). (B) Meta-profiles of hnRNP C and U2AF65 binding at the 3' splice sites of intergenic *Alu* exons (right) and all other non-*Alu* exons present in the same genes (left). Graphs showing the total number of crosslink events on each nucleotide of hnRNP C (blue) and U2AF65 in control (dark green) and *HNRNPC* knockdown (light green) cells. The number of exons in each category is indicated above.

Figure S5

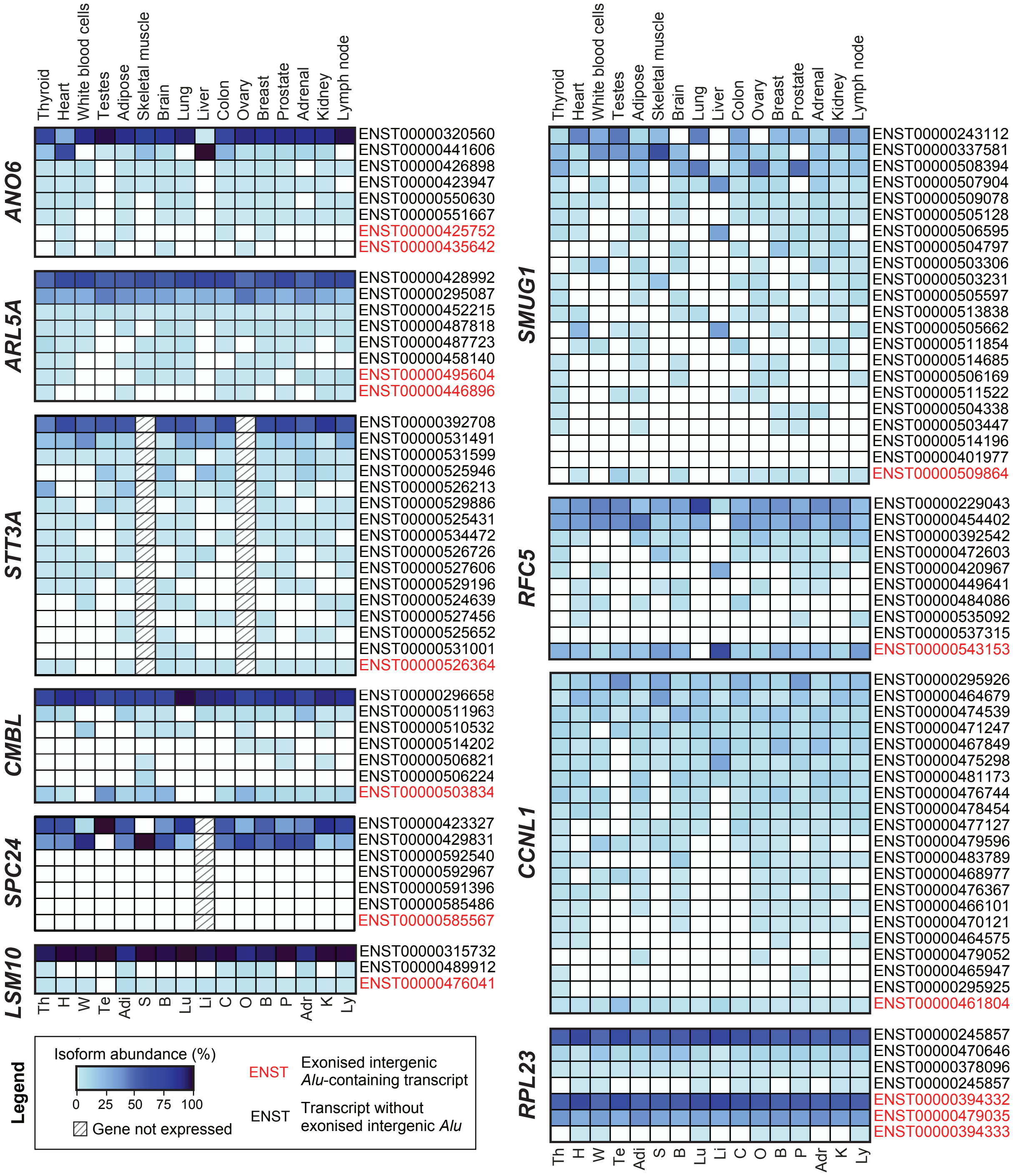

Figure S5 Relative isoform abundances of ten human genes with new transcript 3' ends derived from intergenic *Alu* exons.

Heatmaps showing the relative abundance of all annotated isoforms of ten selected *Alu* exon-containing human genes with mouse orthologues (Figure 4). For each isoform, the relative expression values in each tissue are displayed by increasing shades of blue. 16 different tissues from the Illumina Body Map 2.0 dataset were used: adrenal (Adr), thyroid (Th), heart (H), testes (Te), adipose (Adi), skeletal muscle (S), white blood cells (W), brain (B), lung (Lu), liver (Li), colon (C), ovary (O), breast (B), prostate (P), kidney (K), and lymph node (Ly). Relative expression values are based on the 2-based logarithm of fragments per kilobase per million fragments mapped (FPKM) including a pseudocount of 1 [log2(FPKM+1)]. The *Alu* exon-containing isoforms are indicated in red. Ensembl transcript IDs are given on the right.

Figure S6

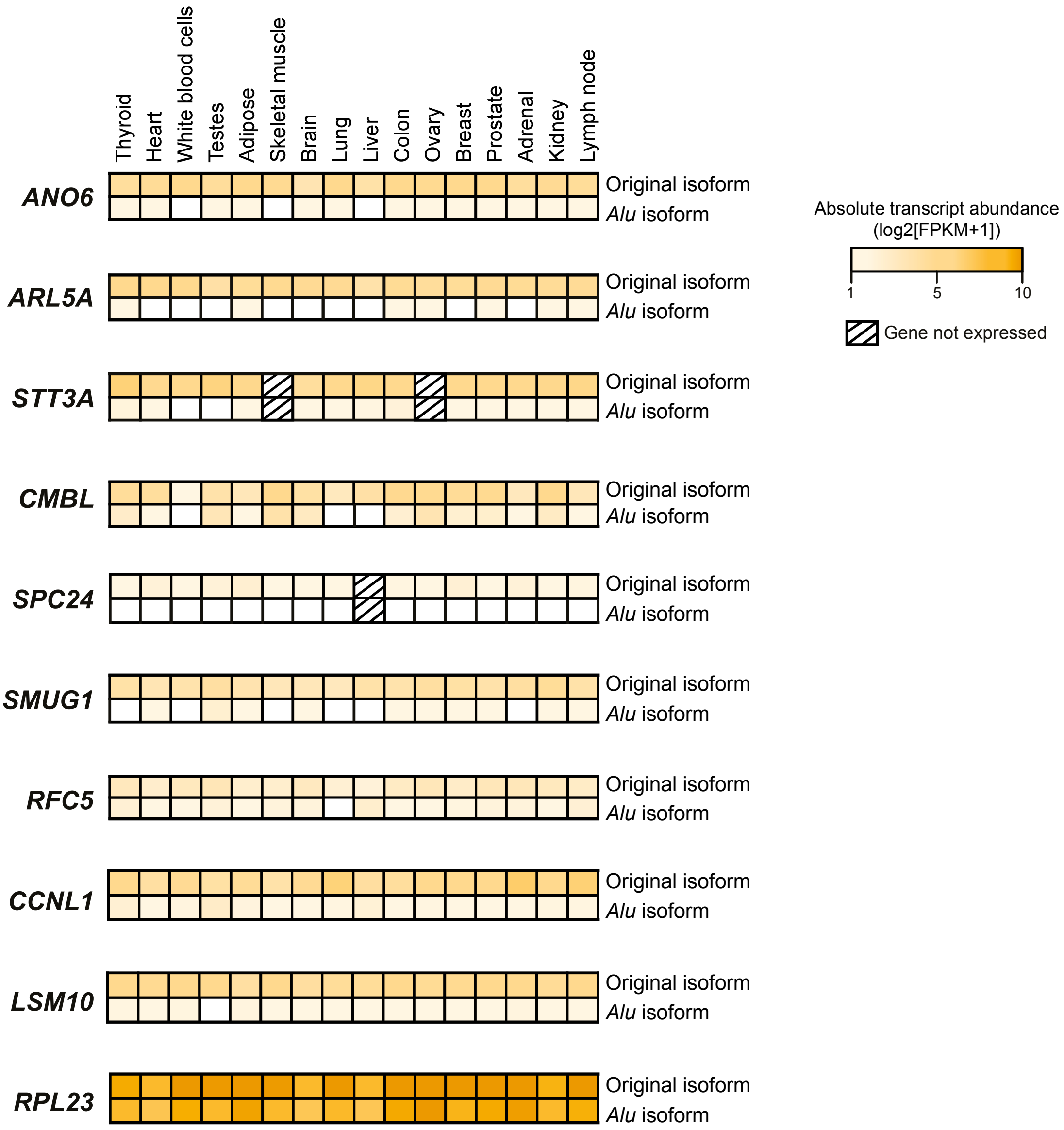

Figure S6 Quantification of absolute isoform abundance levels of the original and *Alu*-derived isoforms across 16 human tissues.

Quantification of absolute abundance levels for ten selected human genes (Figure 4) from the Illumina Body Map 2.0 dataset are shown for the original (top) and for the *Alu* exon-containing isoforms (bottom) in 16 different tissues: adrenal (Adr), thyroid (Th), heart (H), testes (Te), adipose (Adi), lymph node (Ly). Transcripts were grouped as in Figure 4. The values are presented as the 2-based logarithm of the FPKM values including a pseudocount of 1 [log2(FPKM+1)].

Figure S7 (continued on next page)

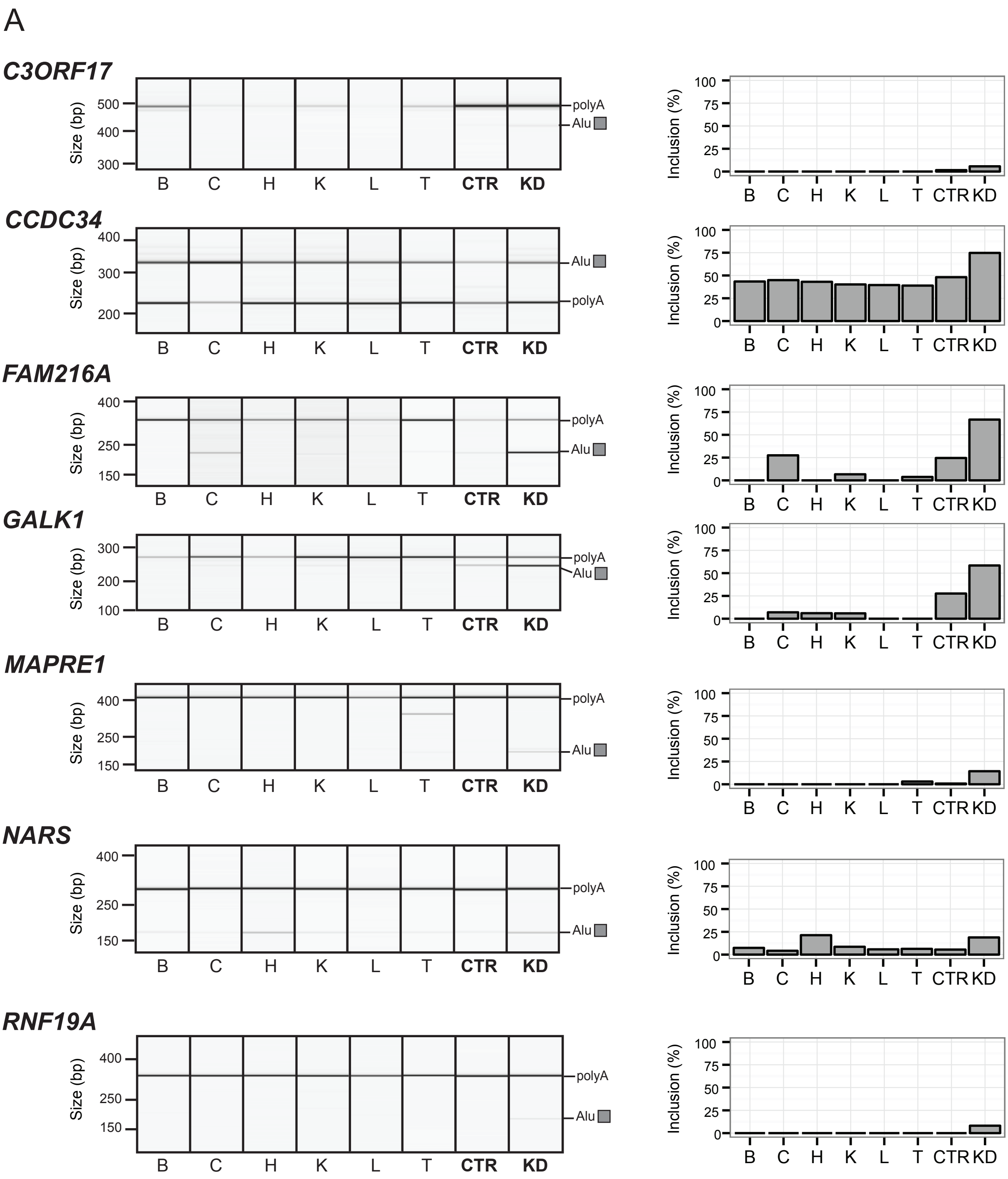

Figure S7 (continued from previous page)

B

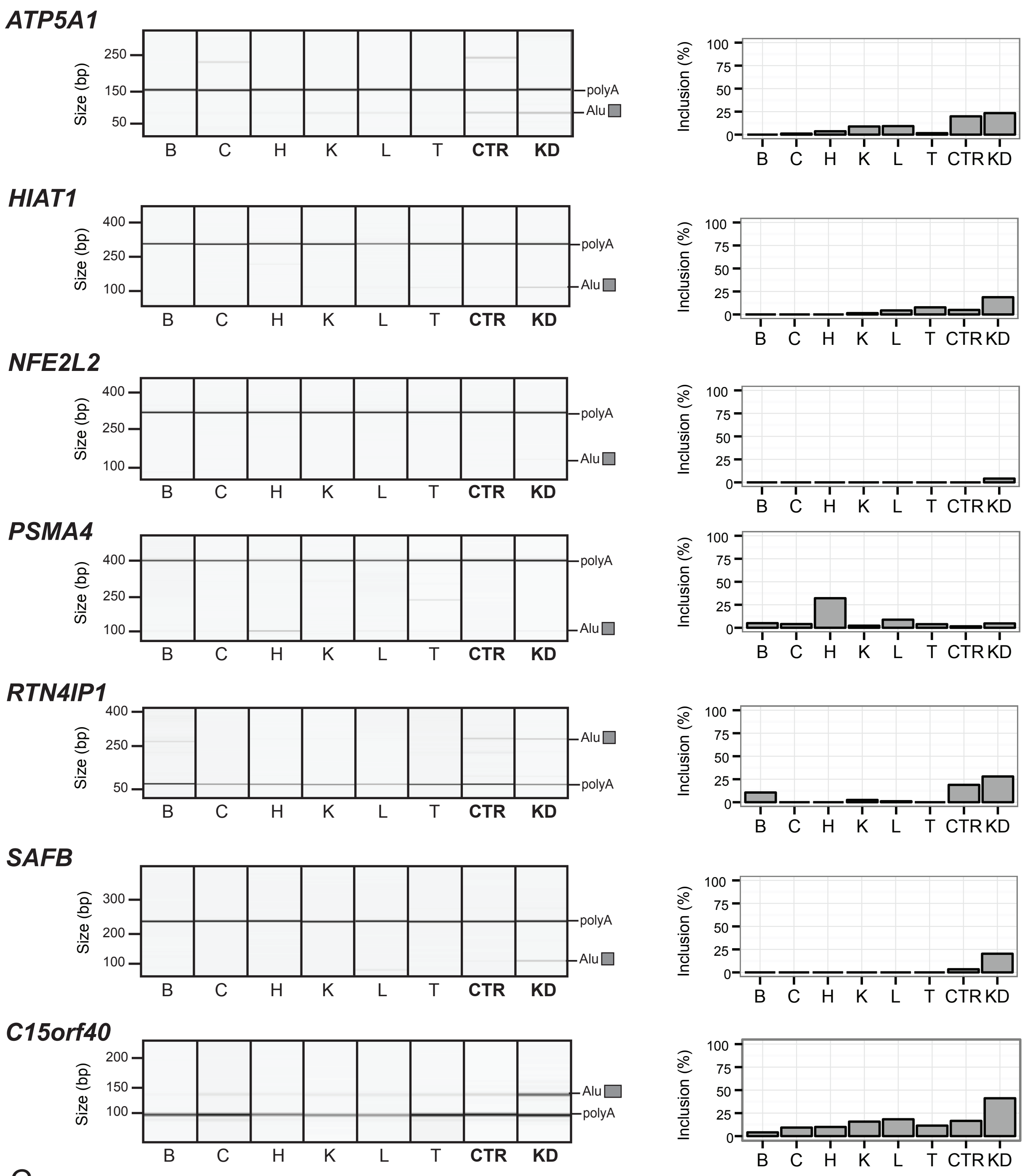

C

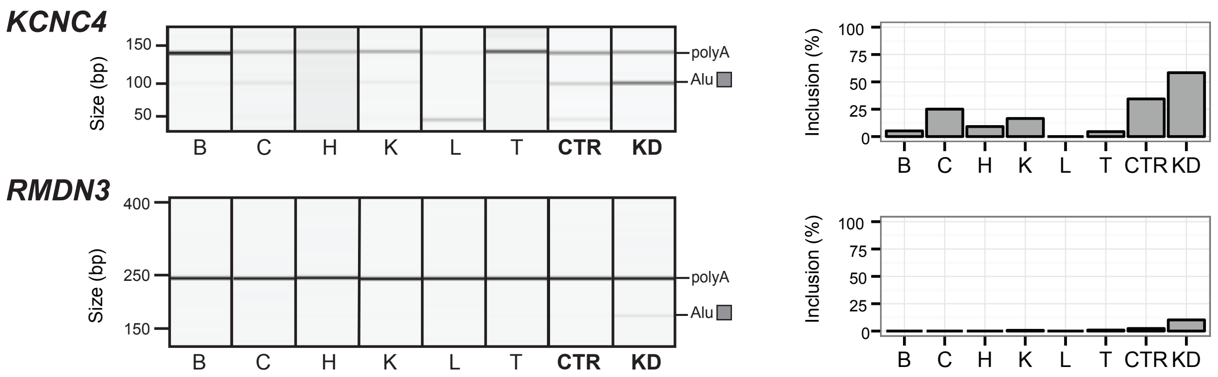

Figure S7 Quantification of intergenic Alu exon inclusion across six human tissues.

Semiquantitative RT-PCR measuring the inclusion of intergenic *Alu* exons that employ (A) cryptic 5' splice site activation, (B) terminal exon skipping, or (C) both scenarios. *Alu* exon inclusion was measured across six different human tissues: brain (B), cervix (C), heart (H), kidney (K), liver (L), testes (T), as well as control (CTR) and *HNRNPC* knockdown (KD=KD1) HeLa cells. Presentation as in Figure S2.

Figure S8

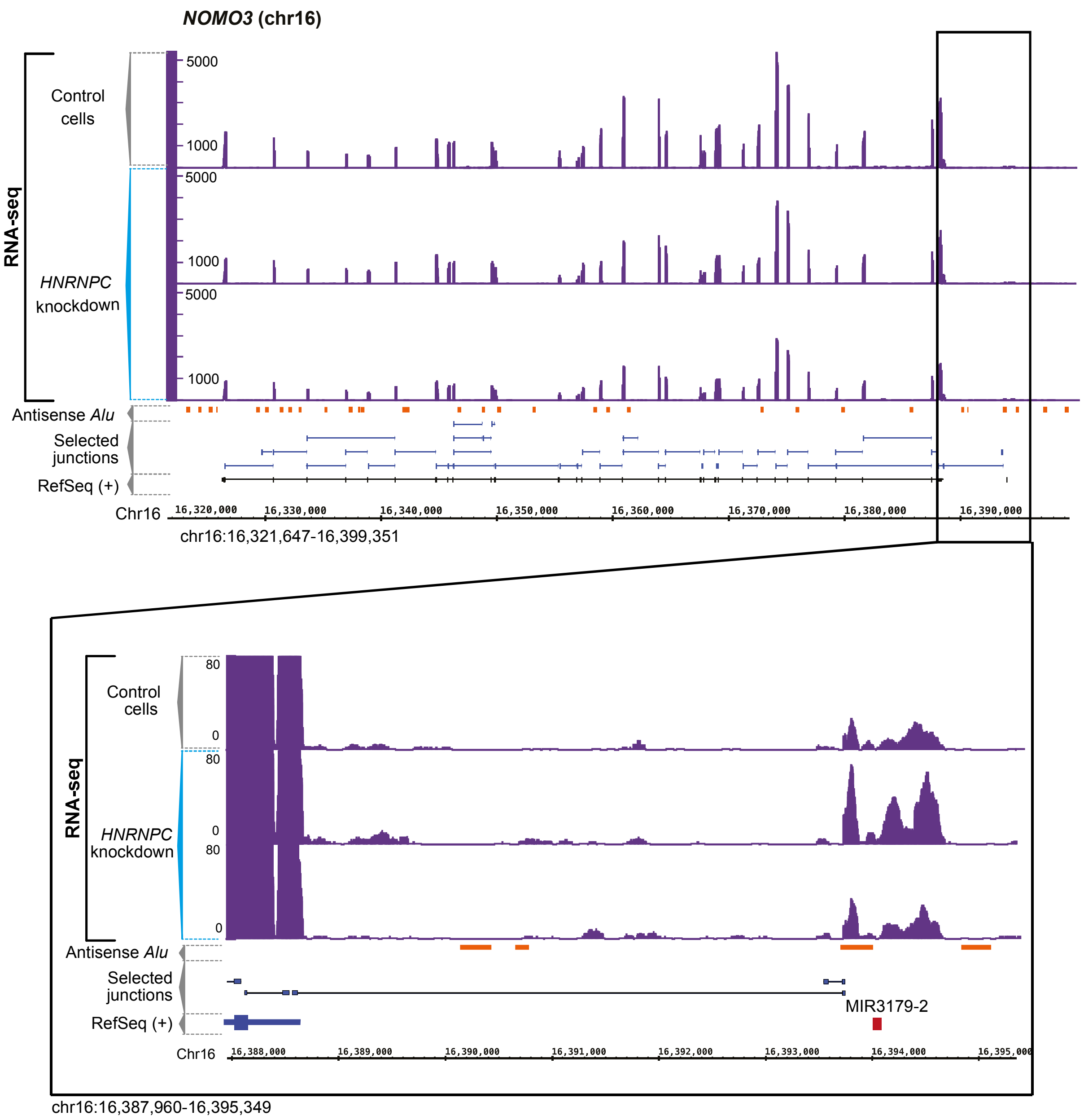

Figure S8 Intergenic *Alu* exonisation might influence miRNA regulation.

Genome browser view of the *NOMO3* gene (chr16, nt 16,321,647-16,399,351, plus strand) presenting the RNA-seq data from control and *HNRNPC* knockdown HeLa cells (labeling as in Figure 1A). Zoom-in view is shown below (chr16, nt 16,387,960-16,395,349, plus strand): the exonisation of the intergenic *Alu* element in absence of hnRNP C via activation of a cryptic 5‘ splice site within the terminal exon that causes the mature transcript to include the miRNA coding region (miR-3179-2), shown in dark red.

Table S1 Overview of the 107 intergenic *Alu* exons.

For each intergenic *Alu* exon, the Ensembl gene ID of the respective gene, the genomic coordinates and width of the intergenic *Alu* exon and its distance from the last genuine polyA site of the gene (in nt) as well as the fold change (log2) in normalised exon expression upon *HNRNPC* knockdown (KD1 over control) are given. The last column specifies the type of the scenario of intergenic *Alu* exonisation.

Gene name	Ensembl gene ID	Genomic coordinates	Width	log2fc (KD1)	Distance from polyA (nt)	Scenario
AFMID	ENSG00000183077	chr17, 76205839-76205919, +	81	-0,17	2058	Cryptic 5'ss
ALDH3A1	ENSG00000108602	chr17, 19636648-19636686, -	39	2,47	4612	Terminal skipping
AMMECR1L	ENSG00000144233	chr2, 128617049-128617155, -	107	0,58	2050	Terminal skipping
ANKRD37	ENSG00000186352	chr4, 186326345-186326373, +	29	-0,5	4951	Terminal skipping
ANP32B	ENSG00000136938	chr9, 100788818-100789130, +	313	0,01	10438	Terminal skipping
ATP1B1	ENSG00000143153	chr1, 169123131-169123220, +	90	1,2	21172	Both
ATP5A1	ENSG00000152234	chr18, 43660866-43663400, -	2535	2,92	711	Terminal skipping
BCL2L2	ENSG00000129473	chr14, 23786151-23786268, +	118	2,12	5184	Terminal skipping
BRCA1	ENSG00000012048	chr17, 41187570-41187665, -	96	-0,04	8648	Cryptic 5'ss
C15ORF40	ENSG00000169609	chr15, 83657081-83657580, -	500	2,54	386	Terminal skipping
C2ORF15	ENSG00000241962	chr2, 99823097-99823496, +	400	1,88	9009	Both
C2ORF49	ENSG00000135974	chr2, 105965708-105965985, +	278	0,57	41	Cryptic 5'ss
C3ORF17	ENSG00000163608	chr3, 112718303-112719068, -	766	1,85	2220	Cryptic 5'ss
CCDC34	ENSG00000109881	chr11, 27359645-27359782, -	138	1,38	295	Cryptic 5'ss
CCDC88A	ENSG00000115355	chr2, 55505823-55506166, -	344	-0,08	8813	Terminal skipping
CECR5	ENSG00000069998	chr22, 17610483-17610565, -	83	1,18	7837	Both
CENPA	ENSG00000115163	chr2, 27019085-27019285, +	201	1,45	1625	Cryptic 5'ss
CES3	ENSG00000172828	chr16, 67009368-67010439, +	1072	2,11	318	Both
CMBL	ENSG00000164237	chr5, 10276337-10276560, -	224	-0,66	2830	Cryptic 5'ss
CPSF4L	ENSG00000187959	chr17, 71242197-71242502, -	306	0,57	2087	Terminal skipping
CSNK1G2	ENSG00000133275	chr19, 1982598-1982713, +	116	1,63	1262	Cryptic 5'ss
DESI2	ENSG00000121644	chr1, 244884477-244884861, +	385	0,78	12143	Terminal skipping
DHX40	ENSG00000108406	chr17, 57687500-57687863, +	364	-0,71	1795	Cryptic 5'ss
DNAJA2	ENSG00000069345	chr16, 46985782-46985858, -	77	0,02	3442	Both
DNAJC19	ENSG00000205981	chr3, 180685823-180690076, -	4254	-0,08	11422	Terminal skipping
DNAJC22	ENSG00000178401	chr12, 49747762-49747796, +	35	0,27	2056	Cryptic 5'ss
DPH7	ENSG00000148399	chr9, 140448337-140448362, -	26	0,2	1000	Cryptic 5'ss
DPY30	ENSG00000162961	chr2, 32236719-32237125, -	407	0,28	11837	Cryptic 5'ss
EXOSC10	ENSG00000171824	chr1, 11122959-11124009, -	1051	0,22	2667	Cryptic 5'ss
EXOSC2	ENSG00000130713	chr9, 133582183-133582961, +	779	1,41	1936	Terminal skipping
FAM160B2	ENSG00000158863	chr8, 21963663-21965985, +	2323	0,33	1432	Cryptic 5'ss
FAM216A	ENSG00000204856	chr12, 110928905-110931055, +	2151	1,17	716	Cryptic 5'ss
FN3KRP	ENSG00000141560	chr17, 80686322-80686450, +	129	0,33	430	Cryptic 5'ss
FZD5	ENSG00000163251	chr2, 208618105-208620094, -	1990	0,51	7217	Terminal skipping
GALK1	ENSG00000108479	chr17, 73747548-73747818, -	271	2,17	142	Cryptic 5'ss
GCLM	ENSG00000023909	chr1, 94348784-94348987, -	204	-0,4	1775	Terminal skipping
GDE1	ENSG00000006007	chr16, 19508120-19508204, -	85	0,18	4812	Cryptic 5'ss

(Continued on next page)

Gene name	Ensembl gene ID	Genomic coordinates	Width	log2fc (KD1)	Distance from polyA (nt)	Scenario
(Continued from previous page)						
GPHN	ENSG00000171723	chr14, 67649582-67649724, +	143	-0,35	1063	Terminal skipping
GRB2	ENSG00000177885	chr17, 73300575-73300826, -	252	-0,69	13332	Terminal skipping
HIAT1	ENSG00000156875	chr1, 100552090-100553216, +	1127	3,48	3158	Terminal skipping
HIST1H2AK	ENSG00000184348	chr6, 27802386-27802421, -	36	-0,16	3238	Cryptic 5'ss
IKBKE	ENSG00000143466	chr1, 206671483-206671514, +	32	2,76	1261	Both
KCNC4	ENSG00000116396	chr1, 110789871-110791048, +	1178	1,1	13206	Both
KNOP1	ENSG00000103550	chr16, 19705484-19714312, -	8829	0,25	591	Cryptic 5'ss
LRRC57	ENSG00000180979	chr15, 42828158-42831809, -	3652	0,25	2912	Terminal skipping
MAMSTR	ENSG00000176909	chr19, 49208608-49210668, -	2061	-0,28	5588	Terminal skipping
MAPKAPK3	ENSG00000114738	chr3, 50708123-50708607, +	485	2,18	21404	Both
MAPRE1	ENSG00000101367	chr20, 31443924-31446713, +	2790	1,92	5714	Cryptic 5'ss
MKRN2	ENSG00000075975	chr3, 12644583-12644806, +	224	0,17	19372	Cryptic 5'ss
MORC2	ENSG00000133422	chr22, 31307285-31307418, -	134	-2,52	15179	Both
MRPS23	ENSG00000181610	chr17, 55904622-55905180, -	559	1,23	11663	Terminal skipping
MSH6	ENSG00000116062	chr2, 48038958-48040516, +	1559	-0,15	4867	Terminal skipping
NAP1L1	ENSG00000187109	chr12, 76427775-76427995, -	221	-0,76	10676	Terminal skipping
NARS	ENSG00000134440	chr18, 55256065-55258410, -	2346	2,18	9479	Cryptic 5'ss
NEURL1	ENSG00000107954	chr10, 105366156-105367169, +	1014	-0,09	13848	Terminal skipping
NFE2L2	ENSG00000116044	chr2, 178091856-178092681, -	826	1,95	357	Terminal skipping
NHP2L1	ENSG00000100138	chr22, 42063344-42063444, -	101	0,61	6491	Terminal skipping
NOMO1	ENSG00000103512	chr16, 14995081-14995207, +	127	NA	5065	Both
NOMO2	ENSG00000185164	chr16, 18504945-18506118, -	1174	1,61	5065	Both
NOMO3	ENSG00000103226	chr16, 16393732-16394926, +	1195	1,48	5065	Both
NSL1	ENSG00000117697	chr1, 212896461-212897442, -	982	1,21	2054	Cryptic 5'ss
PAPSS2	ENSG00000198682	chr10, 89508560-89512786, +	4227	0,44	1099	Both
PCDHB13	ENSG00000187372	chr5, 140598034-140598058, +	25	0,44	1042	Cryptic 5'ss
PDDC1	ENSG00000177225	chr11, 763327-767013, -	3687	0,12	208	Cryptic 5'ss
PIGT	ENSG00000124155	chr20, 44059812-44062481, +	2670	2,68	4929	Terminal skipping
POLR3D	ENSG00000168495	chr8, 22119652-22119794, +	143	1,05	10168	Cryptic 5'ss
POP7	ENSG00000172336	chr7, 100305843-100306152, +	310	0,94	726	Cryptic 5'ss
PSMA4	ENSG00000041357	chr15, 78842850-78844666, +	1817	1,37	1247	Terminal skipping
PTPRS	ENSG00000105426	chr19, 5197749-5198012, -	264	-0,32	7508	Terminal skipping
RFC5	ENSG00000111445	chr12, 118470290-118470423, +	134	2,05	253	Both
RHPN1	ENSG00000158106	chr8, 144469519-144472582, +	3064	5,1	3130	Cryptic 5'ss
RMDN3	ENSG00000137824	chr15, 41025890-41026555, -	666	2,15	1528	Both
RNF19A	ENSG00000034677	chr8, 101268208-101268736, -	529	2,3	553	Cryptic 5'ss
RPL11	ENSG00000142676	chr1, 24023689-24024553, +	865	2,05	775	Cryptic 5'ss
RPL34	ENSG00000109475	chr4, 109554511-109554843, +	333	1,83	2944	Cryptic 5'ss
RTBDN	ENSG00000132026	chr19, 12918801-12919038, -	238	-0,29	17255	Terminal skipping
RTN4IP1	ENSG00000130347	chr6, 107007695-107007964, -	270	2,5	10940	Terminal skipping
S100A16	ENSG00000188643	chr1, 153577604-153578747, -	1144	0,25	616	Both
SAFB	ENSG00000160633	chr19, 5668613-5668690, +	78	0,78	125	Terminal skipping
(Continued on next page)						

Gene name	Ensembl gene ID	Genomic coordinates	Width	log2fc (KD1)	Distance from polyA (nt)	Scenario
(Continued from previous page)						
SBK2	ENSG00000187550	chr19, 56039977-56040735, -	759	0,26	366	Terminal skipping
SEMA4F	ENSG00000135622	chr2, 74926126-74926164, +	39	0,41	16941	Terminal skipping
SGK494	ENSG00000167524	chr17, 26932567-26934273, -	1707	-0,32	2744	Terminal skipping
SLC27A5	ENSG00000083807	chr19, 59003182-59004007, -	826	3,33	5698	Both
SLC45A4	ENSG00000022567	chr8, 142214773-142215062, -	290	2,5	2204	Cryptic 5'ss
SMUG1	ENSG00000123415	chr12, 54559158-54559262, -	105	0,71	15976	Both
SNF8	ENSG00000159210	chr17, 47000089-47000125, -	37	-0,38	6554	Terminal skipping
SNIP1	ENSG00000163877	chr1, 37996657-37996784, -	128	0,67	5359	Cryptic 5'ss
SPIN4	ENSG00000186767	chrX, 62565375-62565450, -	76	0,53	1658	Cryptic 5'ss
TARDBP	ENSG00000120948	chr1, 11089744-11090556, +	813	-1,41	5505	Cryptic 5'ss
TFR2	ENSG00000106327	chr7, 100210122-100212313, -	2192	0,15	5727	Terminal skipping
TIMM17B	ENSG00000126768	chrX, 48737971-48738150, -	180	-0,34	12581	Terminal skipping
TIMM9	ENSG00000100575	chr14, 58875108-58875132, -	25	0	81	Cryptic 5'ss
TMEM177	ENSG00000144120	chr2, 120444329-120444450, +	122	1,18	247	Cryptic 5'ss
TMEM208	ENSG00000168701	chr16, 67272550-67274112, +	1563	-0,6	9370	Terminal skipping
TMEM254	ENSG00000133678	chr10, 81855123-81855163, +	41	-0,18	2811	Terminal skipping
TOR1A	ENSG00000136827	chr9, 132567333-132570313, -	2981	0,38	4911	Terminal skipping
TRIP10	ENSG00000125733	chr19, 6752204-6752606, +	403	0,64	679	Terminal skipping
TRMT13	ENSG00000122435	chr1, 100618561-100618615, +	55	-1,04	2509	Cryptic 5'ss
TRPM4	ENSG00000130529	chr19, 49724862-49725512, +	651	3,35	9772	Terminal skipping
TSC22D4	ENSG00000166925	chr7, 100063296-100063400, -	105	2,69	743	Terminal skipping
UBA3	ENSG00000144744	chr3, 69103514-69103671, -	158	0,09	211	Cryptic 5'ss
USP16	ENSG00000156256	chr21, 30436299-30437890, +	1592	-0,34	9491	Terminal skipping
WDFY1	ENSG00000085449	chr2, 224720236-224720633, -	398	1,3	19432	Both
ZFP90	ENSG00000184939	chr16, 68613718-68613817, +	100	0,77	3771	Terminal skipping
ZNF496	ENSG00000162714	chr1, 247446453-247448594, -	2142	0,85	12121	Terminal skipping
ZNF717	ENSG00000227124	chr3, 75766542-75768428, -	1887	0,53	10685	Terminal skipping
ZNF765	ENSG00000196417	chr19, 53928583-53928713, +	131	1,54	13324	Terminal skipping

Table S2 RT-PCR validation of intergenic *Alu* exonisation events.

Details on semiquantitative RT-PCR confirming increased inclusion of 16 out of 18 intergenic *Alu* exons upon *HNRNPC* knockdown, including 7 cases of terminal exon skipping, 7 cases of cryptic 5' splice site activation, and 2 cases, in which both scenarios were observed (Figures 1E, 1F, S2 and S3) as well of the *Alu* exon-containing gene *CCNL1* from the list of human-mouse orthologues (Figure 4). For each *Alu* exon, the gene symbol and Ensembl gene ID of the respective gene and the genomic coordinates are given, followed by fold change values (log2) in normalised exon expression upon *HNRNPC* knockdown (KD1 or KD2 over control) with associated p-values and percent changes in inclusion from the semiquantitative RT-PCR measurements (KD1 or KD2 versus control). Further, the nucleotide sequences of the used oligonucleotides and the sizes of the expected products (in bp) are indicated (Alu, product indicating *Alu* exon inclusion; pA, product indicating original isoforms).

	**Gene symbol**	**Ensembl ID**	***Alu* exon coordinates**	**RNA-seq log2FC (KD1)**	**RNA-seq log2FC (KD2)**	**RNA-seq p value (KD1)**	**RNA-seq p value (KD2)**	**% PCR change (KD1)**	**% PCR change (KD2)**
**Cryptic 5' splice site activation**	*C3ORF17*	ENSG00000163608	chr3,112718303-112719068, -	1.85	1.09	7.053E-03	1.343E-01	4.05	0.82
	CCDC34	ENSG00000109881	chr11,27359645-27359782, -	1.38	0.80	2.221E-01	3.822E-01	31.51	19.61
	FAM216A	ENSG00000204856	chr12,110928905-110931055, +	1.17	1.10	1.505E-03	1.794E-03	61.74	57.35
	GALK1	ENSG00000108479	chr17,73747548-73747818, -	2.17	1.71	3.415E-05	8.526E-04	29.16	21.90
	MAPRE1	ENSG00000101367	chr20,31443924-31446713, +	1.92	1.11	1.173E-08	5.798E-04	13.20	6.10
	NARS	ENSG00000134440	chr18,55256065-55258410, -	2.18	1.75	0.000E+00	2.206E-11	16.17	8.65
	RNF19A	ENSG00000034677	chr8,101268208-101268736, -	2.30	3.51	1.756E-03	5.112E-05	8.10	7.20
**Terminal exon skipping**	*ATP5A1*	ENSG00000152234	chr18,43660866-43663400, -	2.92	2.11	0.000E+00	2.032E-09	11.03	8.27
	C15ORF40	ENSG00000169609	chr15,83657081-83657580, -	2.54	1.48	6.731E-06	1.138E-02	29.20	23.39
	HIAT1	ENSG00000156875	chr1,100552090-100553216, +	3.48	3.56	8.823E-07	9.103E-07	17.30	15.45
	NFE2L2	ENSG00000116044	chr2,178091856-178092681, -	1.95	0.55	1.532E-05	2.295E-01	2.40	2.20
	PSMA4	ENSG00000041357	chr15,78842850-78844666, +	1.37	0.97	1.996E-02	8.261E-02	2.87	1.87
	RTN4IP1	ENSG00000130347	chr6,107007695-107007964, -	2.50	1.82	4.514E-03	4.682E-02	15.17	15.83
	SAFB	ENSG00000160633	chr19,5668613-5668690, +	0.78	-0.10	NA	NA	12.93	16.01
**Both scenarios**	*RMDN3*	ENSG00000137824	chr15,41025890-41026555, -	2.15	1.14	4.325E-04	8.222E-02	10.10	6.38
	KCNC4	ENSG00000116396	chr1,110789871-110791048, +	1.17	0.24	1.570E-03	4.881E-01	31.04	20.60
**Not validated**	*DNAJA2*	ENSG00000069345	chr16,46985782-46985858, -	0.02	-0.57	NA	NA	N/A	N/A
	CENPA	ENSG00000115163	chr2,27019085-27019285, +	1.45	-0.29	7.102E-02	9.327E-01	0.00	0.00
**Human-Mouse Ortho-logues**	*CCNL1*	ENSG00000163660	chr3,156860663-156864396, -	1.78	2.04	1.777E+00	4.943E-07	13.46	19.60

(Continued on next page)

(Continued from previous page)

Gene symbol	Forward primer	Reverse primer (Alu)	Reverse primer (pA)	Product sizes in bp (Alu/pA)
C3ORF17	ACTTCTTAAAAGCAACCGGC	CTGAGGTCGGGAGTCCAAAA	TTGCATCACCGTCCACGAAT	371/439
CCDC34	AAACCTCGTCCAGCTGCAAA	GTGATTCCAGCTACTCAGGA	GATTGCTCCTGGCTTTATGA	392/242
FAM216A	CAAGATGTAAGTCACTGAAG	GGTCAACAAGAGCATAACTC	ACATGGAACTACATCAGACC	247/338
GALK1	TTTATGGCAGCCGCATGACG	TGTTACCGCACTCCAGACAG	CCGGATATGGAAGATGGCAC	230/275
MAPRE1	GGGGAAAACGACCCTGTATT	AGACCAACCTGCACAGCATA	CTCCAGCATTCAGTGTGACC	201/402
NARS	GGATTGACCCCACTCCCTAT	CCAAGATTGCACTGTTGCAC	CAGAAAGAGAAACCCCAATGA	162/282
RNF19A	TTCCTACATCCCATTGGACA	GAGGCTTGGGCTACAAAGTG	AGGGATGGAGCCTCAAATTC	190/333
ATP5A1	CAAGCCTTGTTGGGCACTAT	GAAGCTGCAGTGAGCTGAGA	GAACAATGACAAAACTGAACTGG	83/152
HIAT1	CTTGGGAACAAACACAAGCC	TGGGAGTTGGAGTCTGTAGT	CCTGGGTGCTGATAGAAAAA	113/285
NFE2L2	TCAGGTAGCCCCTGTTGATT	GTTCCATACCACCCTGGTCA	CTGTCAACTGGTTGGGGTCT	126/293
PSMA4	TGACCTTGAAGTCAGCACTTG	GTGAGACCTTCCCTGCTCAC	AAATGACAAGGACCCAATTCA	104/378
RTN4IP1	ATTGCAGAACTGGTGGATGC	GGAGATCACTTGAGTCTGGG	GGAAGGCTTCTGGAACTTTA	277/78
SAFB	GGCGAGAGAAGCATGTCC	CGCCACTGCACTCTCAGG	GAGACAGGACACAGGATTCCA	108/221
C15ORF40	CCAAGGTCCTAGAACTCAGG	TGTTTGAGCCAGGGAAGTCA	GAGTTGTAGAAGCCAAAAGC	125/93
RMDN3	GATGAAGTTGGCCCTGGAG	ACTCCAGCCTGGGTACAGAA	GGGAGTGGTAGTGGGTAGCA	167/234
KCNC4	GCTCAGCACTGGGGACTATG	TGATGGTATCATTGCACTCCA	TGCCTGTCTCTCTCACGTTG	94/137
DNAJA2	CAACTGGATCAACCCAGACA	TTGTTTGTGTCACTGCACTCC	TGAAGATGTAAAGCTTTGTGGTT	283/411
CENPA	ACCCAGTGTTTCTGTCAGTC	CCAGGAGTTTGAGGCTGCAA	CCTTGGAGTCTCTTTATCTG	171/266
CCNL1	CAGAAGTGCAAGTCGATCGA	ACCTGGGCAACGTAGCAAGA	AAACCAAGAACTGATGCAGG	94/452

Table S3 Gene ontology terms enriched among intergenic *Alu* exon-containing genes.

Summary of Gene Ontology (GO) terms from the three domains Biological Processes, Cellular Components and Molecular Functions that were identified as significantly enriched for the 107 genes with intergenic *Alu* exons using the DAVID Gene Ontology tool. All terms with p-value < 0.05 are listed together with the number of intergenic *Alu* exon-containing genes associated with each term.

Domain	Term ID	Term description	p value	Count
**Biological process**	GO:0034660	ncRNA metabolic process	0.006717106	6
	GO:0000278	Mitotic cell cycle	0.012114501	7
	GO:0006626	Protein targeting to mitochondrion	0.013307169	3
	GO:0070585	Protein localization in mitochondrion	0.013307169	3
	GO:0034470	ncRNA processing	0.015967356	5
	GO:0042254	Ribosome biogenesis	0.02528912	4
	GO:0022402	Cell cycle process	0.026442483	8
	GO:0055085	Transmembrane transport	0.027343007	8
	GO:0007049	Cell cycle	0.046349105	9
	GO:0006839	Mitochondrial transport	0.049585938	3
**Cellular component**	GO:0005829	Cytosol	0.003167032	16
	GO:0043228	Non-membrane-bounded organelle	0.01090679	23
	GO:0043232	Intracellular non-membrane-bounded organelle	0.01090679	23
	GO:0005730	Nucleolus	0.028864531	9
**Molecular function**	GO:0005524	ATP binding	0.004603412	17
	GO:0032559	Adenyl ribonucleotide binding	0.005249821	17
	GO:0030554	Adenyl nucleotide binding	0.008627759	17
	GO:0001883	Purine nucleoside binding	0.00992945	17
	GO:0001882	Nucleoside binding	0.010576759	17
	GO:0015631	Tubulin binding	0.01672496	4
	GO:0000166	Nucleotide binding	0.025971008	20
	GO:0032555	Purine ribonucleotide binding	0.03279343	17
	GO:0032553	Ribonucleotide binding	0.03279343	17
	GO:0017076	Purine nucleotide binding	0.046488926	17

Table S4 Oligonucleotides used for cloning, RT-PCR quantification and 3' RACE of the reporter minigene plasmids.

Identifier and nucleotide sequence are given for all oligonucleotides used for cloning, semiquantitative RT-PCR and 3' RACE of different variants of the *SAFB*, *RMDN3* and *C19ORF60* minigenes (indicated on the left).

Minigene		Oligonucleotide ID	5'-3' sequence
Oligonucleotides used for cloning			
SAFB	**WT**	SAFB_WT_F	TACAAAGCTTTGAAAGCACGTCTGTCTTCCA
		SAFB_WT_R	TACACTCGAGCCGGATGCCTTCCCAGAGGTTTC
	**PPT1**	SAFB_PPT1_F	TTCCTTTATTTAATTATTATTATTATTTTCTTTTTCTTTTGAGATGGAGTCTCGCTCC
		SAFB_PPT1_R	TCTCAAAAGAAAAAGAAAATAATAATAATAATTAAATAAAGGAACAATCAGTGTATATC
	**PPT2**	SAFB_PPT2_F	TAATTCTTATATTCTTAGTAGAGACGGGGTTTCGCCAT
		SAFB_PPT2_R	ACTAAGAATATAAGAATTAGCCGGGTGTGGTGGCAG
	**PAS**	SAFB_PAS_F	TTTTGTAACAAATGTGTTTCCGTTCACATACCC
		SAFB_PAS_R	CACATTTGTTACAAAAAAAAAAAAACCCCAAAACGAAAAACAAATTCACATTG
RMDN3	**WT**	RMDN3_WT_F	TACAAGCTTAGTATCAGGTTTCACTGTTGTCTCT
		RMDN3_WT_R	TGTACTCGAGGACAGGAGCGAAAGAAACTTTG
	**PPT1**	RMDN3_PPT1_F	AATTATAATGTTTTGTTTCTTTCTTTGAGATGGAGTCTCGTTCTGTACCCAGGCT
		RMDN3_PPT1_R	AACGAGACTCCATCTCAAAGAAAGAAACAAAACATTATAATTAAATGGTCTTAAGTGTCC
	**5'SS**	RMDN3_5'SS_F	CATGACTTGATGCCACTATTTAAGGGGGGCGGGGAGGCTTTTTCCTTAGACCTTGC
		RMDN3_5'SS_R	TAAGGAAAAAGCCTCCCCGCCCCCCTTAAATAGTGGCATCAAGTCATGAAGGC
	**PAS**	RMDN3_PAS_F	GAATATATTTTGTTACAAACTTGAAACAAACCAAATTTGATGTTATCAGC
		RMDN3_PAS_R	TAACATCAAATTTGGTTTGTTTCAAGTTTGTAACAAAATATATTCTAGGCAAC
C19ORF60	**WT**	C19orf60_WT_F	TACAAAGCTTGCCCCCTGTCCCGCAGAAAGA
		C19orf60_WT_R	TACAAATGAAGTGAGCGCTGCTGCCCAAGCCGCCTGGCTAGCGGCCGC
	**PPT1**	C19orf60_PPT1_F	TTCTGTTTTATTTTCTTTTATTTTCTTTTAAGACAGGCTCTTGCTCTCTCACCCAGGCTA
		C19orf60_PPT1_R	GTCTTAAAAGAAAATAAAAGAAAATAAAACAGAAAGAAAAAGGCCAAAGAGACGCTGAGA
	**PPT2**	C19orf60_ PPT2_F	CAGGCATAATTCTTGTATTTTGTATTTCTTTTAGTAGAGACGGGGTTGTGCCATATG
		C19orf60_ PPT2_R	CCGTCTCTACTAAAAGAAATACAAAATACAAGAATTATGCCTGTAATCCCAGCTACTC
	**noAlu**	C19orf60_noAlu_F	CTCTTTGGCCGGTACCATACGCGGCCGCGGCCTACTTTTATCCTGGGTCC
		C19orf60_ noAlu _R	AGTAGGCCGCGGCCGCGTATGGTACCGGCCAAAGAGACGCTGAGACGGGT
Oligonucleotides used for RT-PCR quantification			
**All**		pcDNA3_F	ATACGACTCACTATAGGGAGAC
SAFB		SAFB_pA_R	GAGACAGGACACAGGATTCCA
		SAFB_Alu_R	CGCCACTGCACTCTCAGG
RMDN3		RMDN3_Alu_R	ACTCCAGCCTGGGTACAGAA
		RMDN3_pA_R	GGGAGTGGTAGTGGGTAGCA
C19ORF60		C19orf60_R	CTCTGGCGTCTCCATCAACT
Oligonucleotides used for 3' RACE			
**Custom**		QT	CCAGTGAGCAGAGTGACGAGGACTCGAGCTCAAGCTTTTTTTTTTTTTTTTTVN
		Qo	CCAGTGAGCAGAGTGACG
		Qi	GAGGACTCGAGCTCAAGC
RMDN3		RMDN3_GSP1	GCTACAGAGAACTAGGGAAA
		RMDN3_GSP2a	GATGCCACTATTTAAGATGG
		RMDN3_GSP2b	AGATGTCACGAAGGAGATGG
